# Multifunctional Double‐Network Hydrogel with Porous, Adhesive, and Immunomodulatory Properties for Minimally Invasive Soft Tissue Repair

**DOI:** 10.1002/smsc.202500368

**Published:** 2025-10-11

**Authors:** Sara Nejati, Vahid Karamzadeh, Swen Groen, Malvika Nagrath, Luc Mongeau

**Affiliations:** ^1^ Department of Mechanical Engineering McGill University Montreal H3A OC3 Canada; ^2^ Department of Medicine Brigham and Women's Hospital Harvard Medical School Cambridge MA 02139 USA

**Keywords:** curcumin, hyaluronic acid, hydrogels, silk fibroin, tissue engineering

## Abstract

The minimally invasive repair of soft tissue defects remains a major clinical challenge due to the lack of biomaterials that simultaneously fulfill key requirements, including extrudability, strong adhesion, seamless integration, bioactivity, and appropriate mechanical properties. Here, a multifunctional double‐network composite hydrogel is presented that is synthesized from modified hyaluronic acid (HA) and silk fibroin (SF) through a stepwise gelation process. The incorporation of ferric ions enables dynamic crosslinking of dopamine‐grafted HA, resulting in the rapid formation of adhesive hydrogels with microporous structures. Sonication‐induced β‐sheets in SF form a secondary network, enhancing mechanical strength with reduced swelling and degradation. The inclusion of curcumin‐loaded particles within the hydrogel promotes anti‐inflammatory and antifibrotic activity by promoting macrophage polarization toward the reparative M2 phenotype and reducing TGF‐β‐induced fibroblast differentiation and collagen deposition. In situ injectability and printability of the hydrogel are demonstrated in ex vivo porcine vocal fold models. In vitro and in vivo biological evaluations in rat models confirm the cytocompatibility of the hydrogel and its ability to support cell penetration. Mechanical, structural, and biological results collectively support the applicability of this hydrogel as a minimally invasive solution for soft tissue defect repair, particularly in mechanically dynamic tissues such as the human vocal folds.

## Graphical abstract

A multifunctional double‐network hydrogel composed of dopamine‐modified hyaluronic acid, silk fibroin, and curcumin‐loaded particles is developed for minimally invasive soft tissue repair. The hydrogel combines strong adhesion, microporosity, mechanical resilience, and sustained immunomodulatory bioactivity. In vitro and in vivo studies demonstrate its capacity to prevent fibrosis, promote cellular integration, and restore function in dynamic tissues.

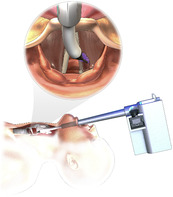

## 1 Introduction

Soft tissue defects arising from disease, trauma, or surgical intervention remain a persistent challenge in clinical practice, often resulting in substantial morbidity and diminished quality of life. Conventional treatment approaches, such as surgical reconstruction and prosthetic implantation, carry risks including donor site morbidity, infection, and suboptimal integration. Minimally invasive strategies using in situ injectable or printable biomaterials offer promising alternatives, with the potential to conform to complex defect geometries, reduce surgical trauma, and enable real‐time adaptation to patient‐specific anatomy.^[^ ^1^, ^2^ ^]^ Clinical applications of this approach have been hampered by the unavailability of materials that simultaneously fulfill multiple criteria, including extrudability for practical delivery, robust tissue adhesion to prevent dislodgement under physiological stresses, microporosity to facilitate cell infiltration and nutrient diffusion, mechanical properties matching those of native tissues, and intrinsic bioactivity to drive tissue repair toward functional regeneration rather than fibrotic scarring. To date, no single hydrogel has successfully integrated all of these attributes for tissue engineering applications.^[^ ^3^, ^4^ ^]^

Hydrogels composed of natural polymers are advantageous for soft tissue repair owing to their intrinsic biocompatibility, tunable properties, and their resemblance to the extracellular matrix. Materials such as collagen, gelatin, chitosan, alginate, hyaluronic acid (HA), and silk fibroin (SF) have been extensively explored as scaffold components. Each natural polymer offers specific tradeoffs between advantages and limitations.^[^ ^5^ ^]^ Of particular interest here, HA, a naturally occurring polysaccharide in connective tissues, stands out for its high biocompatibility, ability to support cell migration and proliferation, and the ease with which its backbone can be chemically modified for tailored functionality.^[^ ^6^, ^7^, ^8^, ^9^ ^]^ Despite its many advantages, HA on its own suffers from poor mechanical strength and rapid enzymatic degradation, which together restrict its durability and functional performance in mechanically dynamic or long‐term tissue repair applications. Meanwhile, SF, a fibrous protein derived from silkworm cocoons, is prized for its mechanical robustness, slow degradation, and structural versatility. However, it lacks cell adhesion motifs and tends to be brittle when used alone.^[^ ^10^, ^11^ ^]^

Recent advances in biomaterials science have introduced various strategies to impart multifunctionality to natural hydrogels. Three approaches have shown particular promise for engineering clinically translatable hydrogel systems. The first involves the chemical modification of the polymer backbone. Functionalization or grafting with bioactive groups can create new crosslinking sites, enhance cell‐material interactions, or introduce novel functionalities. For instance, the grafting of catechol or dopamine onto HA has been shown to enable robust tissue adhesion and dynamic crosslinking through metal‐ligand coordination.^[^ ^12^ ^]^ A second widely used strategy is the construction of double‐network (DN) hydrogels. The integration of a primary soft and ductile network with a secondary stiff and brittle network can enhance properties and overcome the tradeoffs inherent in single‐network systems. This is especially valuable for scaffolds intended for mechanically dynamic tissues.^[^ ^13^, ^14^ ^]^ A third approach centers on the integration of controlled drug delivery systems, such as microparticles or nanoparticles, within the hydrogel scaffold to provide localized and sustained release of bioactive or therapeutic agents, thus modulating the local microenvironment and promoting desirable tissue responses.^[^ ^15^, ^16^, ^17^ ^]^

These three approaches offer complementary mechanisms to enhance both the performance and clinical potential of natural polymer‐based hydrogels. In this work, we integrated all three in the rational design of a multifunctional hydrogel platform based on HA and SF. Specifically, dopamine was grafted onto HA to endow the hydrogel with strong tissue adhesion and enable dynamic crosslinking via ferric‐catechol coordination, resulting in a microporous and self‐healing primary network.^[^ ^18^, ^19^ ^]^ Sonication‐induced β‐sheet formation in SF provided a reinforcing secondary network, leveraging the mechanical advantages of the DN architecture.^[^ ^20^, ^21^ ^]^ Additionally, curcumin‐loaded poly(lactic acid) (PLA) microparticles were incorporated to impart sustained anti‐inflammatory and antifibrotic activity, harnessing curcumin's known ability to promote macrophage polarization toward a regenerative M2 phenotype and inhibit TGF‐β‐driven fibroblast activation and collagen deposition.^[^ ^22^, ^23^ ^]^ The resulting microporous DN composite (MDNC) hydrogel overcomes key translational barriers by facilitating minimally invasive, defect‐conforming delivery, achieving strong tissue adhesion and mechanical integrity, and actively promoting regenerative healing. To our knowledge, this is the first material to combine all essential properties required for in situ repair of vocal fold defects (Figure S1, Supporting Information).

## 2 Results

### 2.1 Design and Synthesis of MDNC Hydrogel

The design of the MDNC hydrogel for minimally invasive, defect‐specific repair in soft tissues such as the vocal folds was guided by the following key criteria: 1) effective immunomodulation and fibrosis prevention, 2) injectability or printability for in situ delivery, 3) strong tissue adhesion for stable integration, 4) microporosity for enhanced cell infiltration and nutrient exchange, and 5) mechanical properties compatible with dynamic soft tissue environments.

To satisfy the first criterion, curcumin, a drug known for its anti‐inflammatory and antifibrotic effects, was encapsulated into PLA particles. PLA is a biocompatible and biodegradable polymer that degrades into lactic acid, a naturally occurring metabolic byproduct, ensuring safe clearance from the body.^[^ ^24^, ^25^ ^]^ Its hydrophobic nature slows curcumin release, enabling a sustained and controlled drug delivery profile. Curcumin‐loaded PLA particles were synthesized and incorporated into the hydrogel network to provide sustained release of curcumin at the injury site, thereby modulating the local immune response favorably toward healing without excessive scar formation.^[^ ^22^, ^26^ ^]^

To address the second, third, and fourth criteria, HA was chemically modified with dopamine groups to enhance its adhesion properties through catechol chemistry, inspired by the adhesion capabilities of marine mussels. This modification could also effectively address HA's intrinsic limitation in cell adhesion, which is crucial for supporting anchorage‐dependent cell functions such as survival, proliferation, morphology, and migration.^[^ ^12^, ^27^ ^]^ The modification of HA was carried out according to protocols established in our previous publication and verified using proton nuclear magnetic resonance (^1^H NMR) spectroscopy (Figure S2, Supporting Information).

Upon the addition of ferric ions, a tris‐complex coordination forms between dopamine in HA and the ferric ions, establishing the first network. This method capitalizes on the inherent chelating properties of the catechol groups present in dopamine, facilitating coordination with ferric ions to form chelates and thereby create micropores. More specifically, such tris‐coordination leads to the emergence of bicontinuous polymer‐rich and polymer‐poor phases. The crosslinking of the polymer‐rich phase, while the polymer‐poor phase mainly comprises water, results in a structure with interconnected open spaces that facilitate cell migration and nutrient transport.^[^ ^28^, ^29^ ^]^ Such dynamic network formation also imparts favorable rheological properties for minimally invasive applications, including injectability and printability. Rapid gelation via ferric‐dopamine coordination confers shear‐thinning behavior, enabling the material to flow readily under stress and rapidly recover its structure upon deposition, which is crucial for accurate, conformal defect filling.^[^ ^18^, ^30^ ^]^

**Figure 1:**
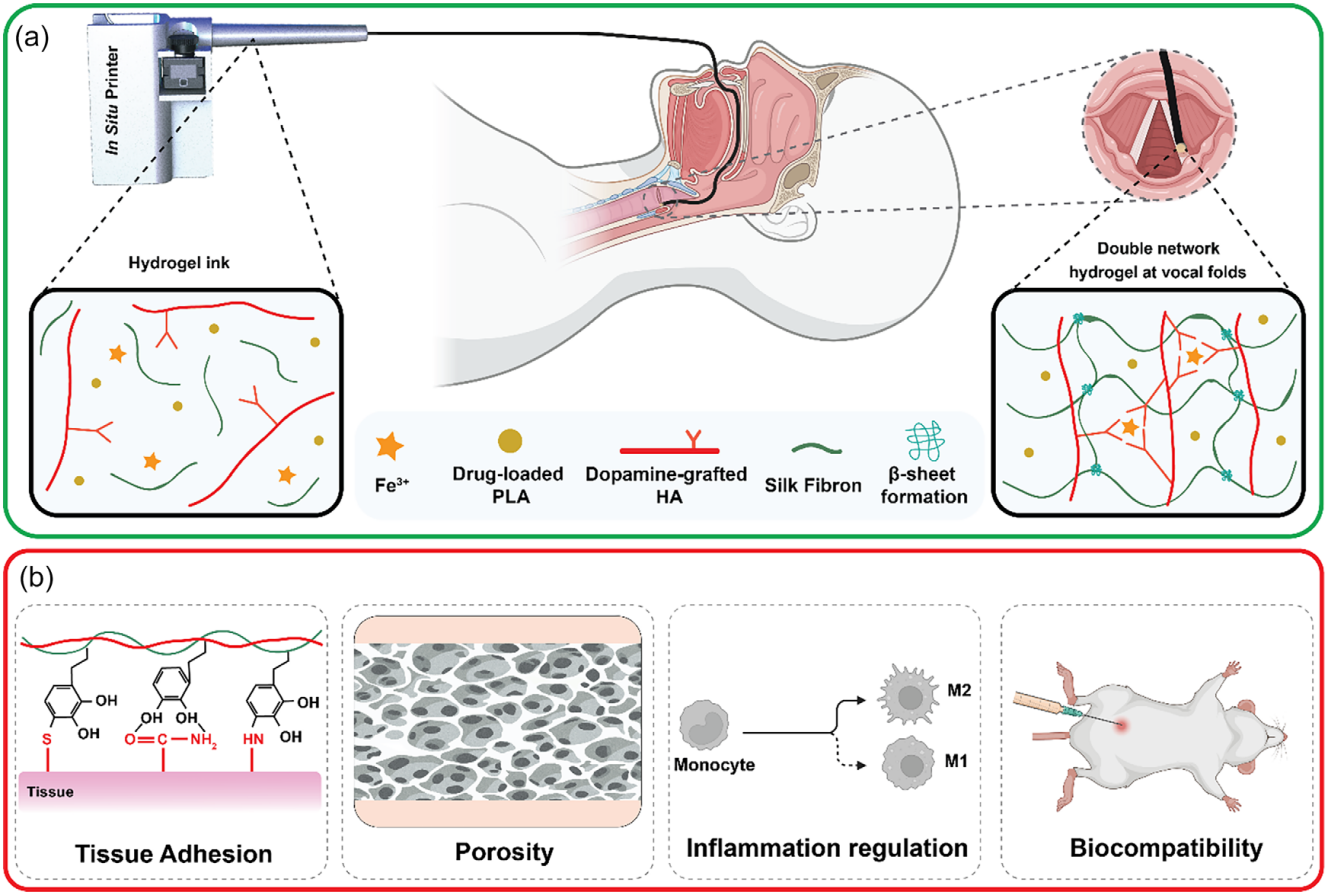
Schematic illustration depicting: a) MDNC hydrogel's components and crosslinking, and b) its multifaceted functionality for in situ vocal fold repair.

To fulfill the final criterion, SF, known for its robust mechanical properties, was incorporated to construct the secondary network. This was achieved by mixing sonicated SF with dopamine‐grafted HA (DAHA) in the presence of ferric ions. The ferric‐dopamine tris coordination rapidly formed the initial dynamic network, thereby adjusting viscosity and shear‐thinning properties to allow smooth injection or printing. Subsequent sonication‐induced β‐sheet formation in SF facilitated the gelation of the secondary network, enhancing the overall mechanical stability and integrity of the hydrogel, reducing degradation rates, and mitigating swelling (**Figure** **1**).^[^ ^31^, ^32^ ^]^

The MDNC was synthesized with an optimized formulation of curcumin‐loaded PLA particles incorporated into a hydrogel precursor consisting of 2.5% SF, 2.5% DAHA, and 10 mM ferric ions. For comparative analysis, nanoporous single network (NSN) hydrogels containing 5% SF, microporous single network (MSN) hydrogels containing 5% DAHA, and microporous DN (MDN) hydrogels containing equal parts of SF and DAHA (2.5% each) were also synthesized.

### 2.2 Particle Size, Loading Efficiency, and Release Profile

Curcumin, known for its potent anti‐inflammatory and antifibrotic properties, was encapsulated in PLA particles to address its inherently low bioavailability. Such encapsulation shields curcumin from degradation while augmenting its solubility and absorption within the body and ultimately enhances its therapeutic efficacy.^[^ ^33^ ^]^

**Figure 2:**
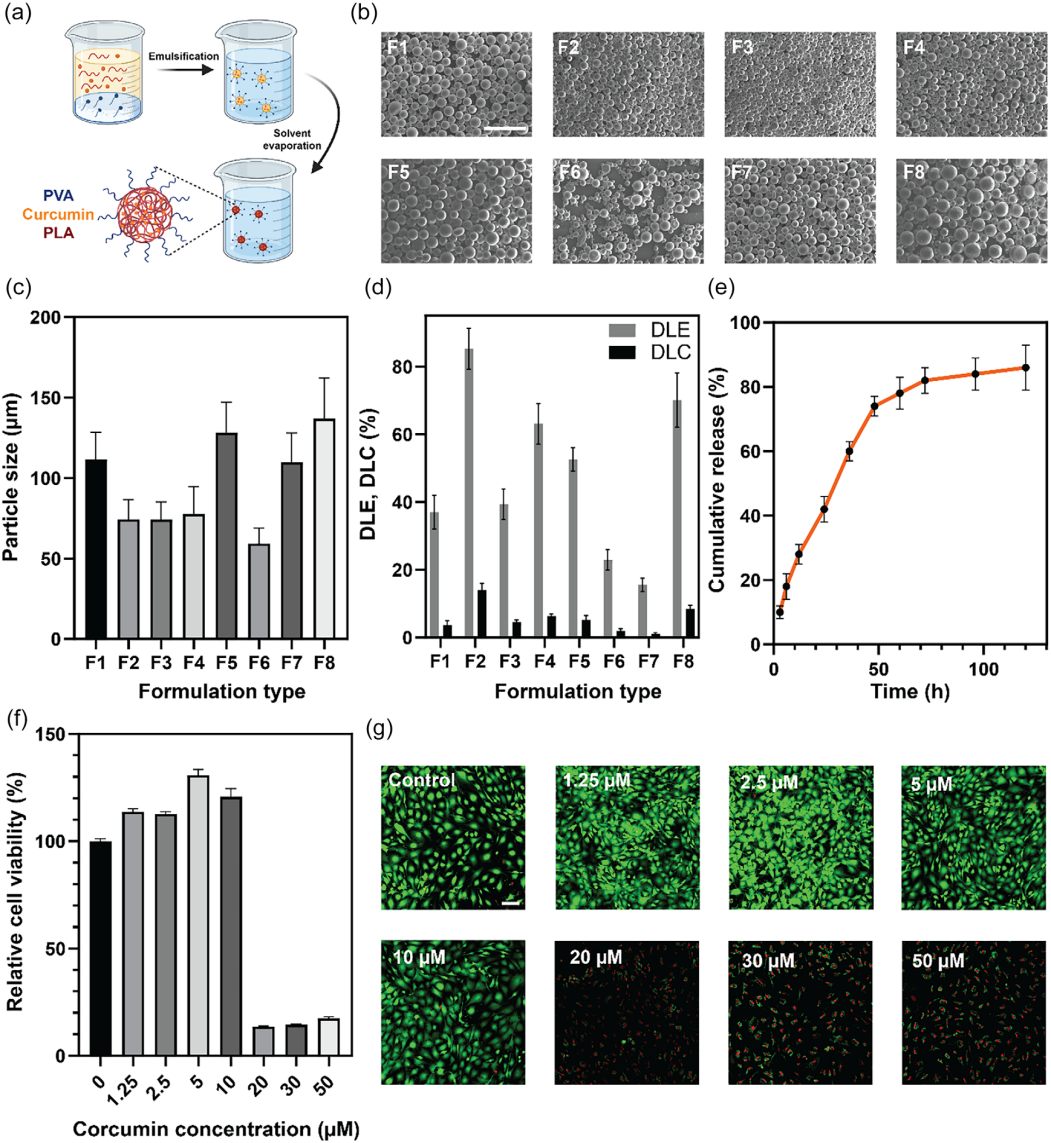
Characterization of curcumin‐loaded PLA particles. a) Schematic representation of the particle fabrication process. b) SEM images of particles fabricated under various formulations (F1‐F8). Scale bar: 500 μm. c) Particle size comparison across different formulations measured using Image J (*n* = 50 particles). d) DLE and DLC of particles for each formulation (*n* = 3). e) Cumulative release profile from the F2 formulation monitored by analysis tube method (*n* = 3). f) Relative cell viability assessed by WST‐1 assay at varying concentrations of curcumin, used to determine the maximum safe concentration for cellular applications (*n* = 3). g) Fluorescent images from live/dead assay, illustrating cell viability at different curcumin concentrations, verifying its maximum safe concentration. Scale bar: 100 μm. Data are presented as mean ± SD.

The encapsulation was carried out using the emulsion solvent evaporation technique, guided by a design of experiment (DOE) approach (**Figure** **2**a). This methodological approach aimed to systematically evaluate the impact of various formulation parameters on particle properties such as size, drug loading efficiency (DLE), and drug loading content (DLC) (Figure S3, Supporting Information). To streamline the experimental process while effectively exploring these relationships, a Taguchi design was implemented, which significantly reduced the number of necessary experiments.

Scanning electron microscopy (SEM) was used to image the particles fabricated under different conditions (Figure 2b). The average particle size was quantified using Image J (Figure 2c). The DLE and DLC of these particles were also determined using a curcumin calibration curve (Figure S4, Supporting Information) and are depicted in Figure 2d. Formulation 2 (F2), with an average diameter of 74.39 μm, and DLE and DLC of 85.69% and 14.02%, respectively, was selected due to its advantageously smaller particle size and superior loading efficiency and content.

The drug release profile of the F2 particles was monitored using the dialysis tube method, as illustrated in Figure 2e. In phosphate‐buffered saline (PBS), these particles released 82% of the encapsulated curcumin over the first 72 h. Such a rapid release profile is crucial for therapeutic efficacy, particularly in the initial phases of the healing process, where the risk of inflammation progressing toward a proinflammatory M1 macrophage phenotype is heightened. By delivering a substantial concentration of curcumin during this critical period, the formulation may promote a shift toward the M2 phenotype, which is associated with anti‐inflammatory responses and facilitates tissue remodeling, thus effectively minimizing the likelihood of fibrotic tissue development.

It is important to note that previous studies have demonstrated that incorporating drug‐loaded microparticles into hydrogel matrices can further prolong drug release compared to particles alone, with the extent of sustained release depending on the hydrogel's porosity and crosslink density.^[^ ^34^, ^35^ ^]^ Based on this body of evidence, we anticipate that our composite hydrogel system would provide at least comparable, if not more sustained, curcumin release relative to PLA particles alone. Our findings confirm that encapsulating curcumin in PLA particles effectively addresses its inherently low bioavailability and solubility, and our in vitro and in vivo assays further support the biological efficacy of the delivered curcumin. Future studies will directly quantify release kinetics from the full hydrogel matrix to further optimize therapeutic performance for specific clinical applications.

The biocompatibility of the curcumin‐loaded PLA particles was confirmed through extensive cytotoxicity testing. The maximum safe concentration of curcumin, determined to be 10 mM, was established using human vocal fold fibroblasts (hVFFs) through WST‐1 and live/dead assays (Figure 2f,g). This concentration was carefully considered when incorporating the particles into the hydrogel network to maintain cell viability and functionality within the repair site.

### 2.3 Structural Properties

A defining characteristic of the MDNC hydrogels is their interconnected microporous architecture, which arises from a coordination‐driven self‐assembly process. Upon introduction of ferric ions or other multivalent metal crosslinkers, rapid coordination occurs with functional groups such as catechol, carboxyl, or imidazole moieties along the polymer backbone. This process initiates rapid gelation and local phase separation, wherein densely crosslinked polymer‐rich regions emerge alongside water‐rich domains.

**Figure 3:**
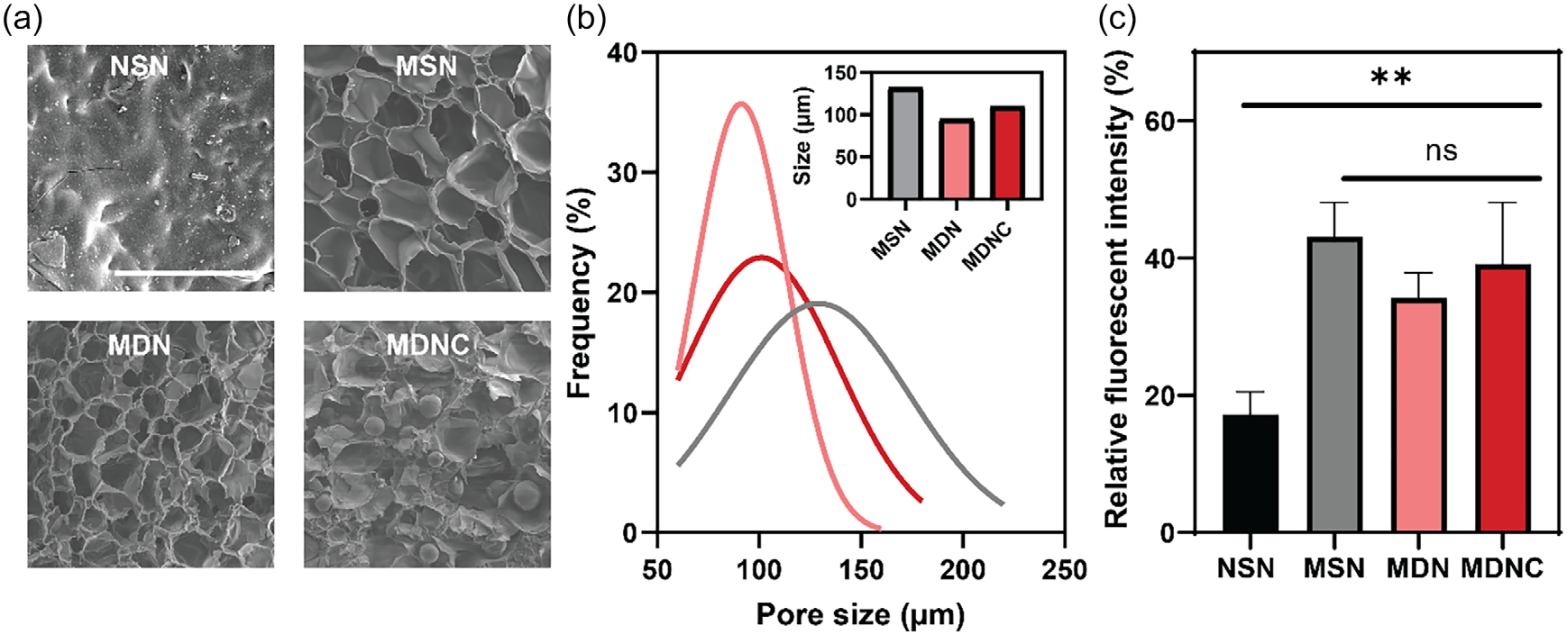
Structural characterization of hydrogels. a) SEM images showing the cross‐sectional morphology of hydrogels dried using CO_2_ critical point dryer (Scale bar = 500 μm). b) Pore size distribution across the hydrogel formulations derived from SEM images (*n* = 50 pores). c) Relative fluorescent intensity of fluorescein dye diffusing into samples, quantifying the permeability and diffusion capabilities of the hydrogels (*n* = 3). Data are presented as mean ± SD. Statistical significance was determined using one‐way ANOVA followed by Tukey's Multiple Comparison Test. ns: not significant; *p < 0.05; **p < 0.01.

To characterize these properties, we synthesized and examined various hydrogel compositions, including NSN, MSN, MDN, and MDNC, using SEM (**Figure** **3**a,b). The samples were dehydrated using a CO_2_ supercritical dryer to minimize artifacts. As expected, the NSN hydrogels displayed no detectable pores. The mesh size of NSN and most existing hydrogels is on the order of 10 nm, which is well below the resolution limit of SEM. In contrast, formulations containing DAHA displayed micrometer‐sized pores, which facilitate cell infiltration and migration effectively. SEM analysis showed average pore sizes of 132.30 μm for MSN, 95.18 μm for MDN, and 110.21 μm for MDNC. These sizes confirm the effective pore‐forming capability of DAHA through tris‐coordination within the hydrogel network. Generally, pores of ≈50–200 μm are considered beneficial for many cell types, including fibroblasts. We also verified the porous structures using confocal microscopy on hydrogels containing fluorescein isothiocyanate (FITC)‐labeled macromolecules in their wet state (Figure S5a, Supporting Information). This process involves no drying or lyophilization treatment.

Recent studies have shed light on the underlying mechanisms governing this phenomenon. For example, Wang et al. demonstrated that the degree of deprotonation and the net charge of metal–organic polyhedra can be tuned to modulate the kinetics and extent of coordination‐driven self‐assembly, ultimately yielding hierarchical colloidal networks with well‐defined microporosity. Their findings further highlight that the resulting network structure, including pore size and mechanical integrity, can be precisely tailored by adjusting the concentrations of metal ions and coordinating ligands, the incorporation of organic linkers, and the sequence of gelation and postsynthetic modifications.^[^ ^36^ ^]^

In a related approach, Shao et al. reported that dual coordination between Fe^3+^ ions and carboxylated cellulose nanofibrils within a poly(acrylic acid) matrix produces a physically crosslinked hydrogel with hierarchical porosity and superior mechanical robustness. In their system, sacrificial hydrogen bonds dissipate energy under stress, while dynamic metal–carboxylate coordination maintains the integrity and reversibility of the network. The presence of multivalent ions strengthens the hydrogel through additional crosslinking and also screens electrostatic repulsions among polyanionic chains, further promoting the association of polymer segments and the emergence of interconnected porous structures.^[^ ^37^ ^]^

These findings from the literature, combined with our own experimental observations, underscore the utility of coordination‐driven phase separation as an effective strategy to generate and modulate microporosity in hydrogel networks. By systematically varying parameters such as the concentration and ratio of metal ions to ligand‐bearing polymers, as well as assembly conditions including pH and sequence of mixing, the size, connectivity, and mechanical stability of the pores could be finely controlled.

The interconnected porous structure of the MDNC hydrogels facilitates superior diffusion, which is critical for fluid transport and mass exchange within the hydrogel and with its surrounding environment. Enhanced diffusion is crucial for supporting the survival, activities, and functionality of cells located in the deeper layers of hydrogels by ensuring adequate delivery of nutrients and oxygen. This characteristic is particularly vital in scenarios where immediate vascularization is absent. To evaluate the diffusion properties, FITC dye was introduced into the hydrogels, and its diffusion was monitored over time using confocal microscopy (Figure S5b, Supporting Information). Images captured after 5 min showed the extent of diffusion, and the relative fluorescent intensity was quantified by converting these images to grayscale (Figure 3c). Our findings indicate that diffusion in MDNC hydrogels is ≈2.2 times greater than in NSN hydrogels. Such increased permeability ensures that cells infiltrating the hydrogel receive adequate nutrients and oxygen, critical for their survival and functionality in nonvascularized environments.^[^ ^38^ ^]^

As swelling affects the physical and mechanical robustness, the swelling behavior of hydrogels was evaluated next. Due to difficulties in accurately measuring the weight of hydrated porous materials, we quantified swelling by monitoring the dimensional changes of hydrogels after immersion in PBS (Figure S6, Supporting Information). The MDNC hydrogels exhibited excellent stability, maintaining their original sizes with less than a 25% size change, indicative of their robust crosslinked network. Such stability ensures that the hydrogels maintain their shape and volume within the tissue environment, avoiding undue compressive stresses on surrounding tissues. The introduction of the secondary SF network significantly mitigated the intrinsic swelling propensity of HA networks. This reduction is attributed to the additional crosslinking provided by β‐sheet formation in SF and the hydrophobic nature of β‐sheets, which limits water absorption.

The biodegradability of MDNC hydrogels was evaluated under enzymatic conditions designed to mimic the physiological environment (Figure S7, Supporting Information). When subjected to lysozyme and hyaluronidase, the MDNC hydrogels exhibited ≈78% mass loss over 28 days. This rate of degradation is well‐suited for vocal fold repair applications, where the scaffold is required to provide temporary support during the critical healing phase. The gradual breakdown of the hydrogel facilitates a progressive transfer of load to the regenerating tissue, thereby supporting tissue growth and integration. Importantly, this controlled degradation process did not compromise the structural integrity necessary for restoring normal vocal fold function.

### 2.4 Mechanical Characterizations

**Figure 4:**
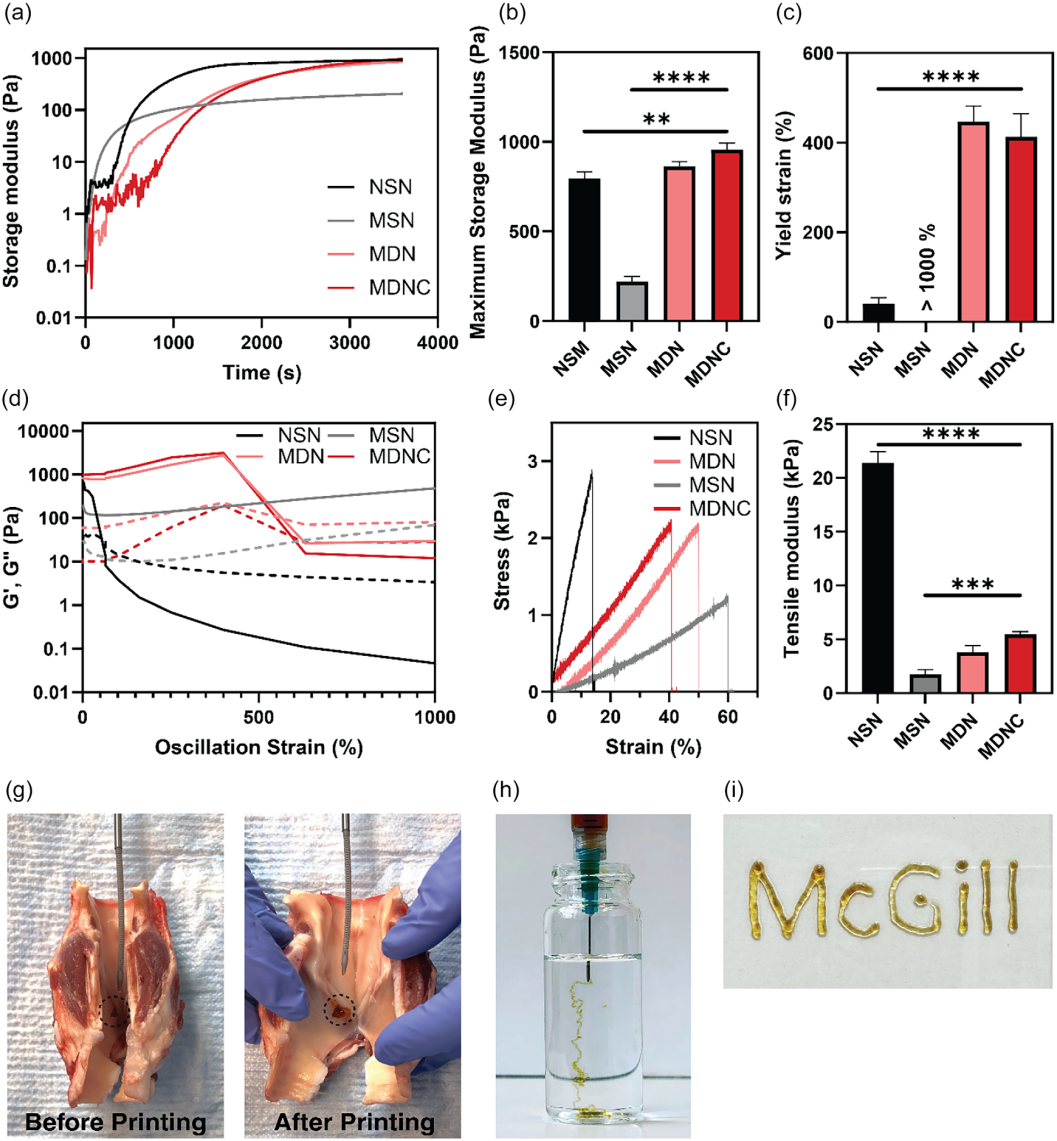
Mechanical and rheological characterization of hydrogels. a) Time sweep test results displaying the storage modulus (G′) and loss modulus (G″) over a 1‐h period, illustrating gelation kinetics. b) Maximum storage modulus among various hydrogel formulations. c) Oscillatory yield strain values across all hydrogel types. d) Amplitude sweep tests presenting G′ and G″ across a strain range of 0.01%−500%, indicating viscoelastic behavior. e) Stress−strain relations illustrating mechanical robustness for different hydrogel formulations. f) Young's modulus for each hydrogel type. g) In situ printability of the MDNC hydrogel in a porcine vocal fold model using an in‐house in situ printer. h) Injectability test results for MDNC in a 0.5% agarose solution using a 23G syringe needle. i) Evaluation of printability using an extrusion‐based 3D bioprinter. Data are presented as mean ± SD (*n* = 3). Statistical significance was determined using one‐way ANOVA followed by Tukey's Multiple Comparison Test. **p < 0.01; ***p < 0.001; ****p < 0.0001.

The dynamic storage modulus (G′) and loss modulus (G″) of our hydrogels were investigated to define their rheological properties across various compositions. Oscillatory time sweep tests produced modulus‐time diagrams, illustrating the gelation process (**Figure** **4**a). The gelation of our double‐network hydrogels involves two processes: initial coordination and subsequent crosslinking. The initial solidification occurs promptly upon mixing, in less than 5 s, through ferric‐dopamine tris coordination, which effectively prevents dilution by body fluids or leaching to unintended areas.^[^ ^19^ ^]^ This is followed by a gradual stiffening process as β‐sheets form in SF over ≈50 min, leading to mechanically stable hydrogels.

The resulting MDNC hydrogels exhibited viscoelastic responses akin to biological tissues. The storage moduli for MDNC hydrogels, at ≈1 kPa, fall within the stiffness range typical of various biological tissues, including vocal folds, lungs, heart, and gastrointestinal tract. The maximum storage modulus of single networks is ≈0.2 kPa for MSN and 0.8 kPa for NSN (Figure 4b).

The modulus‐strain diagrams obtained from oscillatory strain amplitude sweep tests are depicted in Figure 4d. Overall, the G′ values, characterizing stored energy and elasticity, are much higher than those of G″, which characterizes energy dissipation and viscosity. This is typical of highly crosslinked polymeric networks. The yield strain was calculated based on the linear viscoelastic regime (LVE) limit to determine the incipience of failure (Figure 4c). This point indicates the beginning of plastic deformations of the samples. For MDNC hydrogels, G′ reached ≈1000 Pa, with linear behavior maintained up to about 400% strain, and MDN hydrogels exhibited values comparable to MDNC. In contrast, NSN displayed a yield strain of ≈40%, while MSN did not fail even at 1000% strain. The strain sweeps of DN hydrogels displayed a distinctive nonmonotonic response: G′ increased at intermediate strain, then dropped sharply at higher strains. This behavior is attributed to the interplay between physical crosslinking and the DN structure of our gel. At moderate strains, the ferric–catechol coordination and β‐sheet–reinforced silk fibroin likely promote stress‐induced alignment and engagement of reversible physical crosslinks, temporarily increasing network stiffness and thus G′. Once the critical strain is reached, these sacrificial physical bonds dissociate collectively, leading to a rapid drop in modulus as network connectivity is lost. Similar strain‐stiffening followed by yielding has been reported in other DN and physically crosslinked hydrogels, where transient bond breakage underlies this nonmonotonic rheology.^[^ ^39^, ^40^, ^41^, ^42^, ^43^ ^]^ In our system, the dynamic combination of metal–ligand and β‐sheet interactions provides both initial resilience and energy dissipation, resulting in this characteristic stiffening–yielding transition.

Comparisons of Young's moduli, obtained from tensile test (Figure 4e), showed that the Young's moduli values of MDNC are ≈5 kPa, whereas NSN and MSN exhibited values of about 21 and 1.7 kPa, respectively (Figure 4f). Meanwhile, the elongation at break for MDNC was around 40%, compared to 56% for MSN and 14% for NSN (Figure S8 and S9, Supporting Information).

While NSN hydrogels have been shown to be too stiff and brittle for tension‐bearing tissue engineering applications, and MSN hydrogels too soft and ductile for such applications, MDN and MDNC hydrogels have demonstrated intermediate and promising behavior. Their exceptional mechanical properties are attributed to their double‐network configuration, consisting of two interpenetrating polymer networks (IPNs)—one typically rigid and brittle, composed of SF, and the other soft and ductile, made of DAHA. Each network performs distinct but complementary roles under mechanical strain. During the initial deformation or loading phase, the DAHA network readily deforms, dissipating energy through local chain rearrangements. These crosslinks can break or unbind temporarily, enabling the hydrogel to absorb substantial energy, thus mitigating strain effects and localizing damage. Meanwhile, the SF network remains only partially engaged at low to moderate strains, providing baseline structural integrity and preventing immediate collapse. As the applied strain increases, the soft DAHA network undergoes further elongation, absorbing and distributing stress throughout the hydrogel. If the loading exceeds a certain threshold, microcracks may form in the stiffer SF network. However, these localized cracks do not necessarily compromise the entire hydrogel, as the ductile DAHA network can prevent crack propagation by bridging microfractures and thus preventing catastrophic failure. This synergistic interplay results in markedly enhanced overall mechanical performance, providing a balanced combination of structural integrity (from SF) and energy absorption (from DAHA), yielding stiffness and stretchability rarely achieved by conventional single‐network hydrogels.^[^ ^44^ ^]^

We also assessed the injectability and printability of MDNC hydrogels.^[^ ^45^ ^]^ The hydrogels exhibited shear‐thinning behavior, a crucial property for extrusion through syringe needles or printer nozzles. This property was characterized by a continuous decrease in viscosity from a high of 1370 Pa·s at a shear rate of 0.01 s^−1^ to a low of 0.07 Pa·s at 100 s^−1^, as detailed in Figure S10, Supporting Information. To demonstrate practical applications, the hydrogel was printed into a defect created in a porcine vocal fold model using a custom in situ printer developed in our research group. The hydrogel displayed favorable printability, adherence to the defect site, and immediate structural integrity (Figure 4g and Video 1, Supporting Information). The printability of the hydrogel was also evaluated using an extrusion‐based 3D printer (Figure 4i). This testing demonstrated the hydrogel's capability to maintain structural integrity and fidelity during and after extrusion, underscoring its potential for fabricating complex, patient‐specific structures for regenerative medicine applications.^[^ ^46^ ^]^ Injectability was evaluated by injecting the hydrogel into an agarose matrix with a concentration of 0.5% w/v, designed to mimic the rheological properties and water content of soft biological tissues. This setup demonstrated the hydrogel's ability to be smoothly injected through a 23G needle, showcasing its suitability for clinical applications where precise injection into soft tissue is required (Figure 4h). Further validation of injectability was performed by creating a void in a porcine vocal fold model and successfully filling it with hydrogel, as illustrated in (Figure S11, Supporting Information). The excellent injectability and printability of the MDNC hydrogel are attributed to its rheological properties, which ensure that it flows smoothly under pressure and quickly recovers its mechanical strength once deposited. This makes it ideal for in situ injection or bioprinting applications, where precise material deposition and immediate structural integrity are crucial.^[^ ^47^, ^48^ ^]^ Further validation of this delivery strategy using VF replicas, ex vivo larynges, and large animal models will be essential for confirming its functional efficacy, surgical feasibility, and material performance under true in vivo phonatory conditions. These models offer anatomical fidelity and phonatory capacity comparable to the human larynx and thus serve as a crucial bridge between benchtop development and clinical application.

### 2.5 Tissue Adhesion and Self‐Healing

**Figure 5:**
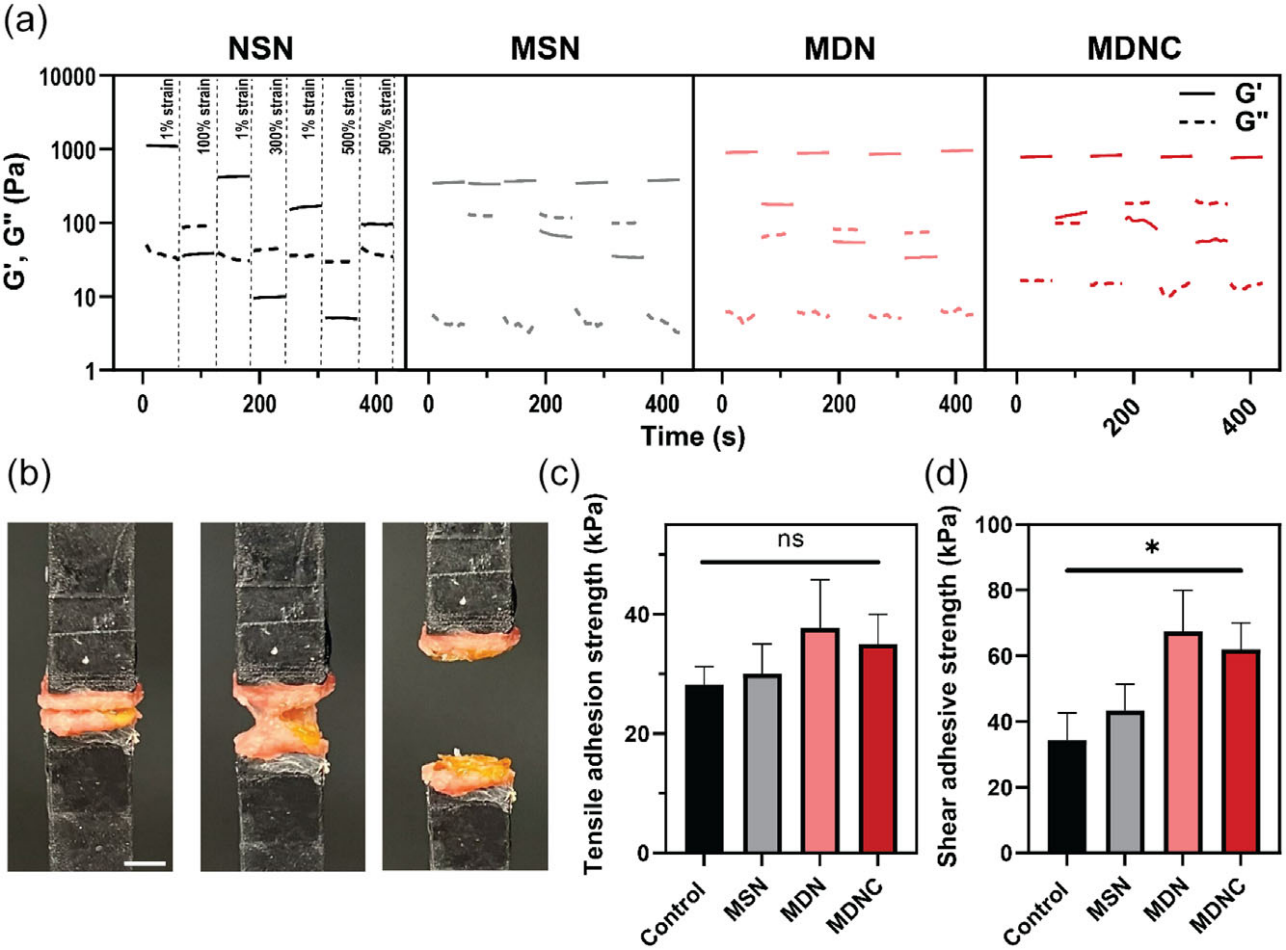
Self‐healing behavior and adhesion strengths of samples. a) Time‐dependent rheological properties during cyclic application of low (1%) and high (100%, 300%, and 500%) strain, showing the self‐healing behavior of NSN, MSN, MDN, and MDNC hydrogels. The solid lines represent the storage modulus (G′), and the dashed lines represent the loss modulus (G″). b) Visual representation of the tensile adhesion test setup using porcine vocal fold tissues (Scale bar = 5 mm). c) Tensile adhesion strength values were measured for the hydrogels against porcine vocal fold tissue. Each bar represents the average strength. d) Shear adhesion strength of the hydrogels against porcine skin. Data are presented as mean ± SD (*n* = 3). Statistical significance was determined using one‐way ANOVA followed by Tukey's Multiple Comparison Test. ns: not significant; *p < 0.05.

The self‐healing capabilities of our hydrogels were evaluated using a combination of oscillatory strain sweeps and time sweeps at periodic low strain (1%) and high strain levels (100%, 300%, and 500%). As shown in **Figure** **5**a, the MDN, MDNC, and MSN hydrogels underwent degradation under increasing oscillatory strain, which induced moduli inversion at each crossover point. Notably, time sweep results demonstrated that these hydrogels exhibited instantaneous healing, regaining their original mechanical strength shortly after stress removal.^[^ ^49^ ^]^ Such self‐healing behavior is attributed primarily to two key mechanisms: 1) the dynamic nature of ferric‐dopamine coordination, and 2) the double‐network configuration.^[^ ^47^, ^50^, ^51^ ^]^ The coordinated bonds formed between ferric ions and dopamine groups act as sacrificial elements, breaking under high strain to absorb and dissipate energy. The reversible and dynamic nature of these coordinate bonds allows them to reorganize and reform rapidly, facilitating the swift restoration of the hydrogel network. In scenarios where the brittle network, primarily composed of SF, fractures under stress, the microfractures are localized due to the presence of the ductile DAHA network. This network can stretch and deform to absorb energy, effectively preventing the propagation of cracks and enhancing the hydrogel's overall toughness and resilience. In contrast, as demonstrated in Figure 5f, the NSN hydrogels did not exhibit self‐healing properties. After being subjected to high strains that led to fracture, the NSN hydrogel failed to restore its original storage modulus. This behavior is attributed to the presence of permanent, irreversible crosslinking within the NSN structure, which does not allow for the reformation of bonds once broken.^[^ ^52^ ^]^

The successful adhesion of delivered hydrogels to host tissue is crucial in medical applications, particularly in areas subject to dynamic mechanical stresses such as the vocal folds. Inadequate adhesion can lead to the dislodgment of the hydrogel from the target site, posing significant risks. For instance, in vocal fold wound filling, a detached hydrogel might obstruct the airways, potentially resulting in fatal outcomes.^[^ ^53^, ^54^ ^]^ Therefore, evaluation of hydrogel adhesion strength is imperative to ensure both safety and efficacy in clinical use.

During phonation, the vocal fold's vibratory motion subjects any microflap wounds to various forces, including tension and shear loading.^[^ ^55^ ^]^ To assess the adhesion strength of our hydrogels under tension, dissected porcine vocal fold tissues were adhered to aluminum blocks (Figure S12a, Supporting Information). Our hydrogel precursor was applied at the tissue interfaces, while fibrin glue, a common surgical adhesive, served as a control for comparison. After allowing adequate time for curing, the bonded assembly was subjected to tension tests using a universal traction test machine (Figure 5b). The adhesive strength was determined by dividing the maximum failure force by the contact area. Our MDNC hydrogel demonstrated an average tensile adhesive strength of 35 kPa, while MDN showed 37 kPa, and MSN showed 31 kPa, all comparable to the control at 28 kPa. The adhesion strength of NSN was too weak to be measured (Figure 5c).

Further assessment of the hydrogels’ adhesive capabilities was conducted through Lap shear tests performed on porcine skin (Figure S12c, Supporting Information). Illustrated in Figure 5d, the MDNC hydrogel exhibited an adhesion strength of 62 kPa, significantly greater than that of the commercial fibrin glue, which has a strength of 34 kPa.

The enhanced adhesion of the MDNC hydrogel is attributed to the inclusion of dopamine in its formulation. Dopamine, inspired by the adhesive proteins of mussels, promotes the formation of strong catecholamine interactions with tissue proteins, facilitating both mechanical interlocking and chemical bonding.^[^ ^56^ ^]^

### 2.6 Cytocompatibility and Cell Migration

**Figure 6:**
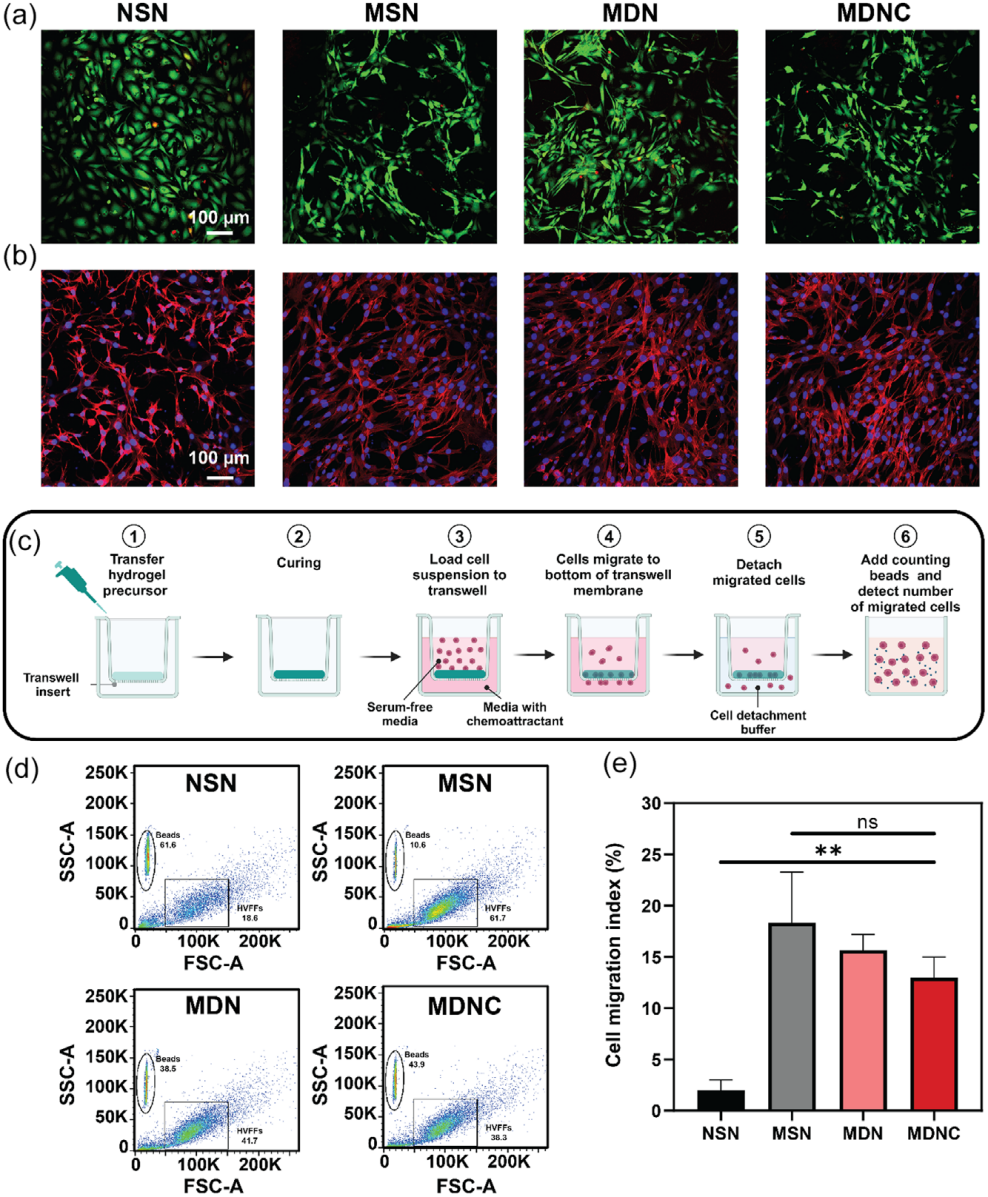
Cell viability, morphology, and migration of hVFFs cultured in hydrogel scaffolds. a) Live/Dead assay images displaying cell viability within NSN, MSN, MDN, and MDNC hydrogel scaffolds after 72 h of culture. Live cells are stained green with Calcein AM, and dead cells are marked in red by ethidium homodimer. b) Confocal microscopy images highlighting cellular morphology. F‐actin filaments are stained red using Alexa 633‐phalloidin, and nuclei are visualized in blue with Hoechst 33 258, indicating active cytoskeletal organization across the different hydrogel formulations. c) Schematic representation of the cell migration assay conducted using a modified Boyden chamber setup to evaluate chemotactic migration of hVFFs through hydrogels. d) Flow cytometry plots categorizing cells and counting beads based on side scatter (SSC‐A) and forward scatter (FSC‐A) properties. e) Average cell migration index for each hydrogel type, quantified from flow cytometry data. Data are presented as mean ± SD (*n* = 3). Statistical significance was determined using one‐way ANOVA followed by Tukey's Multiple Comparison Test. ns: not significant; **p < 0.01.

Hydrogels for tissue engineering must be cytocompatible. We evaluated this property using hVFFs cultured on various hydrogel formulations for three days. Live/dead assays confirmed high cell viability across all hydrogel types, with viability percentages exceeding 85% (**Figure** **6**a).

Cell attachment and cytoskeletal morphology were assessed after three days using rhodamine–phalloidin to stain actin and Hoechst 33 258 to stain nuclei. Figure 6b shows that on NSN hydrogels, cells displayed a roundish and spherical morphology, likely due to the excessive mechanical constraints of the nanoporous matrix and the lack of cell adhesion motifs in the SF, which resulted in poor cell adhesion. In contrast, cells cultured on microporous hydrogels exhibited a healthy, elongated, and fibroblast‐like morphology. This favorable cell behavior is presumably attributed to the microporous structure, which facilitates cell spreading and enhances interactions between dopamine groups and integrin receptors on the cell membrane, thereby improving cell engagement and function.

Cell migration is essential for rapid and effective regeneration in wound healing. The interconnected, micrometer‐scale porosity of our hydrogels supports this process by allowing cell penetration and migration within the 3D scaffold architecture. We utilized a Boyden chamber assay to evaluate the migration of hVFFs through the hydrogels under a chemoattractant gradient over a seven‐day period. The cell migration index was defined as the ratio of the number of cells migrated through the porous membrane to the total number of cells initially added (Figure 6c).

Results indicated that cells effectively penetrated the porous matrices of MSN, MDN, and MDNC hydrogels. In contrast, very few cells migrated into NSN, which lacks sufficient porosity for effective cell migration. After the migration period, cells were collected, combined with counting beads, and analyzed via flow cytometry (Figure 6d). The migration indices recorded were 4.7% for NSN, 16.3% for MSN, 15.5% for MDN, and 19.3% for MDNC, highlighting the superior performance of microporous hydrogels in supporting cell migration (Figure 6e).

Given its outstanding mechanical, structural, and biological properties, MDNC hydrogel was selected for further biological investigations. This decision underscores MDNC's enhanced functionality in promoting cell recruitment and migration, essential for tissue engineering and regenerative medicine applications.

### 2.7 Anti‐Inflammatory and Antifibrotic Properties

Profibrotic cytokine TGF‐β1 is upregulated during various stages of wound healing. It drives fibroblast differentiation into myofibroblasts, which subsequently produce excessive Col I and contribute to scar tissue formation and fibrosis. Such fibrotic remodeling alters the extracellular matrix (ECM) composition and biomechanics, leading to permanent changes that can compromise vocal fold flexibility and phonation quality. In this study, we hypothesized that curcumin could mitigate the profibrotic effects of TGF‐β1. To test this, hVFFs were treated with TGF‐β1 in the presence or absence of curcumin, and the outcomes were evaluated through gene expression and immunofluorescence staining for α‐SMA and Col I.^[^ ^57^ ^]^

**Figure 7:**
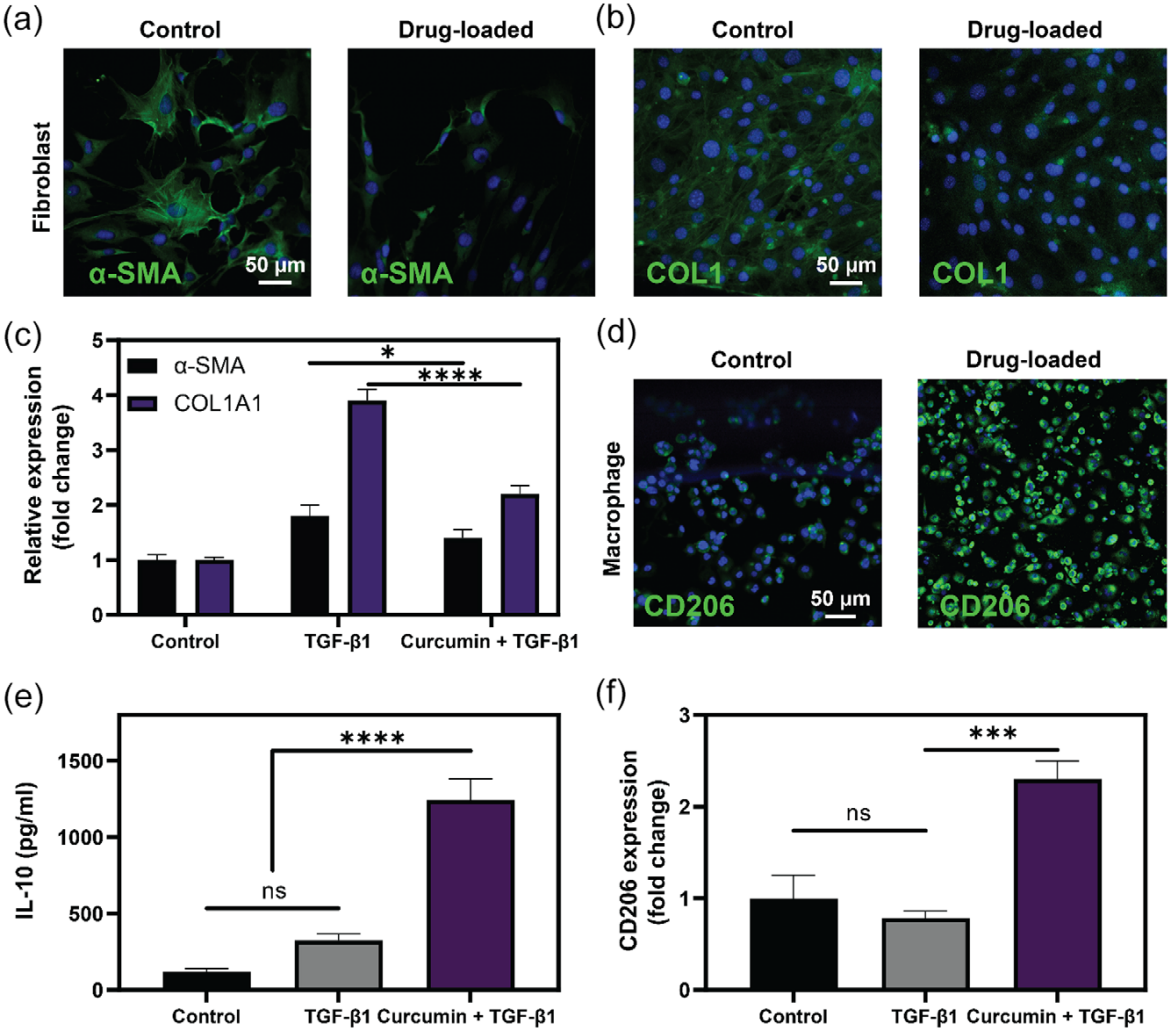
Antifibrotic and anti‐inflammatory effects of curcumin. a) Immunocytochemical analysis of α‐SMA expression in hVFFs after 72 h, with α‐SMA filaments in green and nuclei in blue. b) Immunocytochemical visualization of Col I production in hVFFs, showing collagen fibers in green and nuclei in blue. c) Relative gene expression of α‐SMA and Col I in hVFFs across different treatment groups. d) Immunofluorescence staining of M2 macrophages, highlighting CD206 expression (green) and nuclei (blue). e) ELISA quantification of IL‐10 secretion by macrophages after 72 h. f) CD206 gene expression analysis in THP‐1‐derived macrophages, indicating enhanced M2 polarization following curcumin treatment. Data are presented as mean ± SD (*n* = 3). Statistical significance was determined using one‐way ANOVA followed by Tukey's Multiple Comparison Test. ns: not significant; *p < 0.05; ***p < 0.001; ****p < 0.0001.

Immunofluorescence staining revealed a substantial decrease in both α‐SMA and Col I intensity (**Figure** **7**a,b), with segmented quantification showing a twofold reduction in Col I deposition in the curcumin‐treated group relative to TGF‐β1 stimulation alone (Figure S13, Supporting Information). Gene expression results further confirmed these findings. TGF‐β1 stimulation alone caused a significant upregulation of α‐SMA and COL1A1 gene expression, indicative of enhanced myofibroblast differentiation and ECM production. Fibroblasts treated with TGF‐β1 showed a 1.8‐fold increase in α‐SMA and a 3.9‐fold increase in COL1A1 expression compared to untreated controls. Conversely, fibroblasts treated with TGF‐β1 in the presence of curcumin displayed a 1.2‐fold decrease in α‐SMA expression and a 1.7‐fold reduction in COL1A1 expression compared to TGF‐β1 treatment alone (Figure 7c).

These findings suggest that curcumin effectively mitigates the profibrotic effects of TGF‐β1 by modulating fibroblast activation and ECM production. The antifibrotic mechanism of curcumin may involve its ability to inhibit TGF‐β1 signaling pathways, including Smad2/3 phosphorylation, which are known to drive myofibroblast differentiation.^[^ ^58^ ^]^ Additionally, curcumin's antioxidant and anti‐inflammatory properties may further contribute to its protective effects by mitigating oxidative stress and reducing the autocrine amplification of TGF‐β1 signaling.^[^ ^59^, ^60^ ^]^ By attenuating myofibroblast activity and limiting excessive collagen deposition, curcumin presents a promising therapeutic strategy to prevent fibrosis and preserve the biomechanical properties of the vocal folds, leading to an efficient fibrosis‐free tissue repair.

The investigation of curcumin's anti‐inflammatory potential involved assessing its effect on macrophage polarization. Macrophages play a critical role in wound healing, not only by defending against infections but also by orchestrating tissue repair processes. M2 macrophages, in particular, are crucial for their anti‐inflammatory and tissue remodeling activities that promote healing.^[^ ^61^, ^62^ ^]^ In the current study, THP‐1‐derived macrophages were treated with curcumin, and cytokine secretion, gene expression, and polarization markers indicative of the M2 phenotype were assessed using enzyme‐linked immunosorbent assay (ELISA), quantitative polymerase chain reaction, and immunocytochemistry, respectively.

The results demonstrated that THP‐1‐derived macrophages exhibited a significant shift toward the M2 phenotype when cultured with curcumin‐loaded hydrogels. Immunofluorescence labeling with CD206 antibodies highlighted an increased expression of this M2 marker in the presence of curcumin, compared to the control group (Figure 7d, Figure S14, Supporting Information). This phenotypic shift was further confirmed by assessing morphological differences (Figure S15, Supporting Information) and quantified by measuring interleukin‐10 (IL‐10) concentrations in the culture supernatant. ELISA demonstrated that IL‐10 levels were 3.8 times higher in curcumin‐treated samples than in those without curcumin, indicating a potent anti‐inflammatory response (Figure 7e). Similarly, curcumin treatment led to a 2.9‐fold increase in CD206 expression compared to untreated samples (Figure 7f). These findings highlight the effectiveness of curcumin‐loaded hydrogels in steering macrophage activity toward a phenotype that supports tissue repair and regeneration, underlining the therapeutic potential of curcumin in tissue engineering and regenerative medicine applications.

### 2.8 In Vivo Assessment

**Figure 8:**
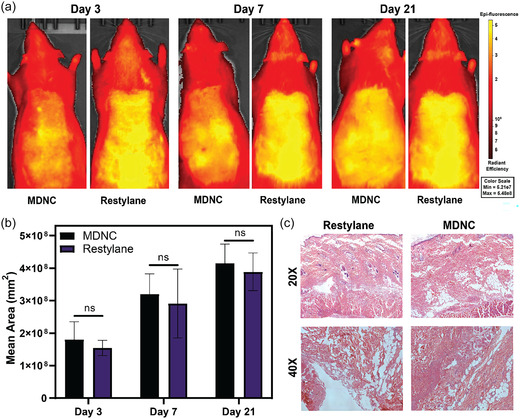
In vivo evaluation of MDNC hydrogel compared to commercial Restylane. a) In vivo fluorescence imaging using ProSense 680 to assess cathepsin activity at various time points. b) Quantification of fluorescence signal areas from in vivo imaging. c) H&E staining of representative sections from subcutaneous injections at day 21 postinjection. Data are presented as mean ± SD (*n *= 3). Statistical significance was determined using one‐way ANOVA followed by Tukey's Multiple Comparison Test. ns: not significant.

The in vivo biocompatibility of MDNC hydrogels was assessed and compared to Restylane, a widely used commercial injectable for soft tissue augmentation. The evaluation was conducted over a 21‐day period following subcutaneous injections into Sprague Dawley rats. Biocompatibility was quantitatively assessed using the In Vivo Imaging System (IVIS), which allowed for the noninvasive monitoring of inflammation via fluorescence imaging. Specifically, ProSense 680, a fluorescent probe sensitive to cathepsin activity often associated with inflammation, was used to track the inflammatory response at the sites of hydrogel injection (**Figure** **8**a). The mean fluorescence areas for both MDNC and Restylane (used as control) were measured on days 3, 7, and 21 postinjection. Results shown in Figure 8b indicated no significant differences between the inflammatory responses of the MDNC hydrogel and Restylane at any time point. This suggests that the MDNC hydrogel does not induce a greater inflammatory reaction than Restylane, supporting its biocompatibility.

To evaluate tissue regeneration capabilities, histological analyses were performed 21 days postinjection on tissues treated with MDNC hydrogel and Restylane. The MDNC hydrogel exhibited greater cellular infiltration and integration with host tissue than Restylane, which displays a more distinct demarcation between the implant and surrounding tissue. This improved integration is largely due to the microporous structure of the MDNC hydrogel, which promotes cell migration and trafficking within the matrix. The foreign body response, typically marked by the presence of inflammatory cells at the material's periphery, was notably reduced in MDNC‐treated tissues. This reduction is attributed to the sustained release of curcumin from hydrogel, which modulates the local immune response to favor healing processes over fibrotic outcomes.

## 3 Discussion

Despite recent advances in biomaterials science, no single hydrogel simultaneously meets all essential criteria for minimally invasive vocal fold repair. Over the past decade, a wide array of injectable hydrogels has been investigated for applications ranging from tissue regeneration and augmentation to scar mitigation and restoration of vocal fold biomechanics. While many of these systems meet some targeted features, such as injectability, matrix mimicry, or cell compatibility, none have successfully integrated the complete set of functional requirements within a single platform. In the following section, we compare the MDNC hydrogel against representative peer‐reviewed systems reported within the past decade, evaluating performance across five critical parameters: 1) extrudability for minimally invasive delivery; 2) tissue adhesion for positional stability; 3) microporosity to support cellular infiltration and nutrient exchange; 4) mechanical compatibility with the native lamina propria; and 5) intrinsic bioactivity to attenuate inflammation and fibrosis. This comparison highlights the benefits conferred by the adopted polymer modification strategy, dynamic and DN architecture, and integrated bioactive delivery.

### 3.1 Natural Hydrogels and ECM‐Derived Systems

HA–based fillers have long been used clinically due to their biocompatibility and ease of injection. Crosslinked HA gels, such as Restylane and Juvederm, are readily extruded through fine needles and elicit only mild inflammation. However, conventional HA injectables lack microporosity, typically resorb within four to six months, and require repeated administration. They tend to migrate within tissue, lack antifibrotic properties, and offer no inherent tissue adhesion. Their mechanical tuning is limited, and although viscoelastic, they rarely match the dynamic modulus of the vocal fold lamina propria. Various strategies, including methacrylation, particle size modulation, and incorporation of reinforcing agents, have been employed to enhance the mechanical integrity and longevity of HA‐based fillers. However, these modifications do not fully address their core limitations in tissue adhesion, microporosity, bioactivity, and biomechanical matching to the vocal fold lamina propria.^[^ ^6^ ^]^

Collagen‐based injectables were among the earliest used for VF augmentation but have declined due to immunogenicity. Gelatin‐based hydrogels emerged as safer alternatives. For example, Ng et al. developed an injectable genipin‐crosslinked gelatin hydrogel that exhibited viscoelastic properties comparable to commercial HA fillers.^[^ ^63^, ^64^ ^]^ Swelling was minimized to reduce airway obstruction risk. However, these hydrogels lack microporosity, tissue adhesion, and bioactivity, and require up to 20 min to fully set, which is significantly slower than the near‐instantaneous gelation of MDNC.

Decellularized ECM hydrogels derived from VF tissue retain key bioactive factors such as decorin and growth factor–binding domains, offering promising intrinsic activity. In a 2020 study, injection of a decellularized VF matrix hydrogel significantly attenuated fibrosis and enhanced tissue repair by suppressing TGF‐β1–mediated myofibroblast differentiation.^[^ ^65^, ^66^ ^]^ These materials are biocompatible and needle‐deliverable but typically lack microporosity and mechanical robustness. Without secondary crosslinking, they degrade rapidly and lack positional stability. While they may outperform MDNC in terms of innate bioactivity, their poor structural integrity and limited retention hinder their application in dynamic environments such as the VFs.

A more recent strategy combined silk fibroin microparticles with crosslinked HA to form an FDA‐approved composite hydrogel for VF augmentation. Here, silk particles provided mechanical strength and slow degradation, while HA ensured injectability. Carroll et al. demonstrated safe delivery and tunable viscoelasticity, but the matrix remained dense and inert, lacking tissue adhesion, porosity, or bioactive cues.^[^ ^67^, ^68^, ^69^ ^]^ While superior to plain HA fillers in longevity, this formulation falls short of MDNC in supporting regeneration or integration with host tissue.

In efforts to replicate the dynamic biomechanics of the VFs, elastomeric protein‐based hydrogels have also been explored. Li et al. introduced an injectable hydrogel composed of resilin‐like polypeptides that crosslink rapidly in situ and exhibit elasticity comparable to native VF mucosa.^[^ ^70^, ^71^ ^]^ These hydrogels demonstrate excellent biocompatibility with minimal fibrotic response. However, they lack microporosity, adhesive functionality, and bioactivity beyond the inherent properties of the polypeptide backbone.

### 3.2 Advanced Microstructured Hydrogels

Microporous annealed particle (MAP) hydrogels, developed by Pruett et al., offer a structurally advanced yet biologically inert platform for vocal fold repair.^[^ ^72^, ^73^ ^]^ Made entirely of PEG‐based microgels (≈50–100 μm), MAP is injected as a slurry and covalently annealed in situ to form a microporous scaffold. The interstitial pores support cell infiltration, nutrient diffusion, and vascularization. Tuned to match vocalis modulus (≈5–6 kPa), the implant remained stable for over six months and promoted native‐like ECM deposition by 14 months. Despite these structural advantages, MAP lacks intrinsic bioactivity, adhesive properties, and degradable natural components.

Mohammadi et al. employed gelatin methacrylate (GelMA) and hyaluronic acid methacrylate to create injectable DN microgels (≈30–50 μm) for VF regeneration.^[^ ^57^ ^]^ These UV‐crosslinked microgels, loaded with the anti‐inflammatory cytokine interleukin‐10 (IL‐10), anneal postinjection into a cohesive, microporous scaffold that supports cell infiltration and matrix remodeling. HA mimics native ECM, while GelMA provides mechanical support, and sustained IL‐10 release attenuates fibrosis. This system meets key criteria, including injectability, microporosity, partial mechanical matching, and bioactivity. However, like many photocured platforms, it depends on synthetic photoinitiators and lacks rapid in situ setting or adhesive functionality.

Bao et al. developed a mechanically robust, injectable pore‐forming double‐network (PDN) hydrogel composed of chitosan and glycol‐chitosan.^[^ ^14^ ^]^ Designed for high endurance, it withstood over 6 million vibration cycles at 120 Hz without failure. Controlled phase separation during gelation produced interconnected pores, enabling cell perfusion through hydrogel samples up to six cm long in a vocal fold bioreactor. While lacking drug delivery or adhesive features, glycol‐chitosan provided inherent antimicrobial and cell‐supportive properties.

Zou et al. developed a dynamic, self‐fusing hydrogel of carboxymethyl chitosan (CMCS) and alginate, crosslinked through reversible Schiff base bonds and photo‐curing.^[^ ^74^ ^]^ Designed to match vocal fold elasticity, it maintained volume and function in a rabbit glottic insufficiency model, outperforming HA fillers. Notably, it induced collagen I/III and elastin production without growth factors, demonstrating intrinsic ECM‐inductive bioactivity. While not overtly microporous, its fibrous network supports nutrient diffusion and cell ingress, and mild tissue adhesion was achieved via dynamic covalent bonding.

In summary, recent progress between 2015 and 2025 reflects clear progression toward multifunctional hydrogel systems engineered to address the complex demands of VF tissue repair. Positioned at the forefront of these innovations, the MDNC hydrogel integrates structural integrity, mechanical compliance, and biological functionality within a single, integrated platform. Table S1 (Supporting Information) synthesizes these advancements and highlights the relevance and competitiveness of MDNC among other state‐of‐the‐art materials.

## 4 Conclusion

The present study introduced a multifunctional DN hydrogel, designed for minimally invasive soft tissue defect repair. This hydrogel demonstrated adhesive strengths comparable to commercially available adhesives such as fibrin glue, ensuring stable placement during tissue repair. Its microporous structure facilitated rapid diffusion and effective cell penetration, which is crucial for effective tissue integration. The double‐network configuration provided superior mechanical behavior. The sustained release of curcumin modulated the local immune response by promoting macrophage polarization toward the reparative M2 phenotype. It also counteracted TGF‐β1‐induced collagen production and fibroblast differentiation into myofibroblasts, thereby preventing fibrosis. In vivo assessments confirmed the favorable performance of our composite hydrogel in terms of tissue compatibility and integration by promoting cellular infiltration and reducing inflammatory reactions. Given its unprecedented combination of mechanical, structural, and biological properties, the proposed material is expected to impact the repair and regeneration of various tissues, particularly mechanically dynamic tissues such as the human vocal folds. While these results are promising, further evaluation in anatomically and functionally relevant models is required to fully validate clinical utility and establish superiority over standard fillers.

## 5 Experimental Section

5.1

5.1.1

##### 5.1.1.1 Fabrication and Characterization of Drug‐Loaded Particles

Curcumin‐loaded PLA particles were synthesized via an oil‐in‐water (O/W) emulsion solvent evaporation. Curcumin and PLA were dissolved in dichloromethane (DCM) to create the organic phase. Polyvinyl alcohol (PVA) was dissolved in water to form the aqueous phase. The organic phase was added to the aqueous phase at a 1:25 volume ratio under continuous mechanical stirring and stirred for 3 h to facilitate DCM evaporation and particle hardening.^[^ ^75^ ^]^

Statistical methods were utilized to establish the influence of factors such as PLA concentration (3% and 6%), PVA concentration (2% and 4%), PVA molecular weight (9 and 23 kDa), and drug concentration (0.3% and 0.6%) (**Table** **1**).^[^ ^76^ ^]^ Postevaporation, microspheres were collected, washed, and air‐dried overnight under a bench with continuous airflow.

**Table 1:** Fabrication parameters for each particle formulation.

Formulation	PLA Con. [%]	PVA Con. [%]	PVA Mw. [kDa]	Drug Con. [%]
1	3	2	9	0.3
2	3	2	23	0.6
3	3	4	9	0.6
4	3	4	23	0.3
5	6	2	9	0.6
6	6	2	23	0.3
7	6	4	9	0.3
8	6	4	23	0.6

Particle size was assessed using a scanning electron microscope (SU3500 variable‐pressure SEM, Hitachi) after Pt coating. DLE and loading content (DLC) were quantified using the equations below, based on plate reader (SYNERGY, BioTek Instrument) measurements calibrated with a standard curcumin curve. The release profile of the optimized formulation was evaluated using the dialysis tube method. To ascertain the maximum safe concentration of curcumin for human vocal fold fibroblasts (hVFFs), cells were treated with various concentrations of curcumin, with viability assessed via live/dead and WST‐1 assays after 24 h.

(1) $$\text{DLE}\left(\%\right)=\frac{\text{Weight of drug in particles}}{\text{Weight of the feeding drug}}\times 100$$

(2) $$\text{DLC}\left(\%\right)=\frac{\text{Weight of drug in particles}}{\text{Weight of particles}}\times 100$$

##### 5.1.1.2 DAHA Synthesis and Hydrogel Fabrication

DAHA was synthesized using a previously established method.^[^ ^77^ ^]^ In brief, HA (1.0 g) was dissolved in 100 mL of deionized water, to which 0.5 g of sodium periodate was added. The mixture was stirred for 5 h in dark conditions and terminated by adding 1 mL of ethylene glycol. Subsequently, the mixture was dialyzed against deionized water and lyophilized, resulting in the oxidized polymer, aldehyde‐modified HA (AHA).

Dopamine was grafted onto AHA through a Schiff base reaction. AHA (1 g) was dissolved in 100 mL of PBS (pH 5.0), followed by the addition of 1 g of dopamine hydrochloride. The solution was mixed for 10 h at room temperature. The reaction was completed by adding 50 mg of sodium borohydride, stirring for an additional 30 min. This solution was then dialyzed against deionized water for three days and lyophilized for three more days to obtain DAHA. Grafting success was confirmed using proton NMR spectroscopy.

For the MDNC hydrogel preparation, 5% DAHA and 5% SF solutions were prepared in deionized water. The SF solution was sonicated at 30% amplitude for 1 min to induce β‐sheet formation in SF. The solutions were then mixed in equal volumes, and drug‐loaded particles were incorporated at a concentration of 50 mg mL^−1^ of the hydrogel mixture. Finally, FeCl_3_ was added to achieve a final concentration of 10 mM, initiating the gelation process (**Table** **2**).

**Table 2:** Composition of each hydrogel formulation.

Hydrogel	Hydrogel precursor
NSN	5% SF
MSN	5% DAHA + 10 mM Fe^3+^ ions
MDN	2.5% SF + 2.5% DAHA + 10 mM Fe^3+^ ions
MDNC	2.5% SF + 2.5% DAHA + 10 mM Fe^3+^ ions + 50 mg/ml curcumin‐loaded PLA particles

##### 5.1.1.3 Structural Characterizations

The polymer network was imaged using an SEM. To maintain native morphology, samples were dried using a CO2 supercritical point dryer (CPD300, Leica). The dehydrated samples were coated with 5 nm Pt using a high‐resolution sputter coater (ACE600, Leica) to increase surface conductivity.

Macro‐ and microscopic pores were also imaged using a confocal microscope (LSM 710, Zeiss). Both HA and SF were conjugated with FITC fluorescent labels according to published protocols. Hydrogel samples were prepared by mixing components in a vial and transferring ≈150 μL into a 35 mm Petri dish with a coverslip bottom (P35G‐0‐10‐C, MatTek). Hydrogels were immersed in PBS and imaged as prepared.

The permeability of our hydrogel samples was assessed using confocal fluorescence microscopy. Cubical samples, 5 × 5 × 5 mm, were prepared and placed in an observation chamber. A 10 mM solution of FITC was introduced as the diffusion medium. Fluorescence images were acquired over a fixed plane 200 μm above the base of the hydrogel, every 10 s over a 5 min period. The images were converted to grayscale using ImageJ, and the mean fluorescence intensity within a defined region of interest was quantified for each time point. The extent of FITC diffusion into each hydrogel was compared by analyzing the relative fluorescence intensity after 5 min.

The swelling ratios were determined by immersing the hydrogel disks (10 mm in diameter, 5 mm in thickness) in PBS (pH = 7.4) at 37 °C with gentle mechanical stimulation (75 rpm). The diameter of the disks was measured using a caliper after 72‐h incubation. The swelling ratio was calculated by dividing the measured diameter size by the initial value. For degradation assays, all hydrogel samples were prepared with a uniform volume of 500 μL. The average dry weight of pristine hydrogels was used as the weight at day 0. An enzyme degradation environment was simulated by adding an enzyme solution containing 13 μg mL^−1^ lysozyme and 100 U mL^−1^ hyaluronidase in PBS to the gels. The samples were incubated at 37 °C with gentle mechanical stimulation over 28 days. The enzyme solution was changed every other day. At predetermined time intervals, the enzyme solution was removed. The samples were then washed three times for 5 min with PBS. The samples were then lyophilized, and the remaining polymer dry weight was measured. The remaining fraction of the polymer mass was calculated by dividing the dry weight of the remaining polymer and the dry weight of the initial gels.

##### 5.1.1.4 Mechanical Characterizations

Rheological measurements were performed using a torsional rheometer (HDR‐2, TA Instruments) equipped with parallel plates (20 mm upper plate diameter). During the time sweep tests, hydrogel samples were subjected to a constant strain of 0.1%. The storage (G′) and loss (G″) moduli were monitored for 1 h at a frequency of 1 Hz. Amplitude sweep tests were conducted by varying the strain from 0.01% to 500% at a constant frequency of 1 Hz to assess the hydrogels’ response to different strain levels. All rheological tests were performed at a constant temperature of 37 °C.

The mechanical strength of the hydrogels was evaluated under ambient conditions using an electronic universal testing machine (Instron 3360, Instron Corporation). Rectangular samples measuring 50 mm in length, 10 mm in width, and 2 mm in thickness were prepared. To ensure equilibrium hydration, the hydrogels were immersed in PBS for 24 h prior to testing. Tensile testing was conducted with a 1 N load cell at an elongation rate of 10 mm min^−1^ until the samples broke. The stress–strain curves obtained were analyzed to determine the Young's modulus from the slope of the linear region, ultimate tensile strength from the peak stress before failure, and elongation at rupture from the strain at the point of breakage.

To characterize the shear‐thinning behavior of the MDNC hydrogel precursor, its viscosity was systematically measured across a range of shear rates from 0 to 100 s^−1^. This rheological assessment is needed for its evaluation for printing and injection applications.

To simulate the injectability of the hydrogel into soft tissue, an agarose gel model was utilized. Agarose was dissolved in PBS at a concentration of 0.5% w/w and heated to 95 °C. The solution was cast into glass vials with an internal diameter of 2 cm and cooled to room temperature. The gels were left for 1 h at 37 °C before use. The MDNC hydrogel precursor was then injected into these agarose gels using a 23G hypodermic needle to mimic the clinical scenario of hydrogel delivery into a tissue site.

The printability of the MDNC hydrogel was assessed using a 3D bioprinter (BioAssemblyBot, Advanced Solutions) equipped with a 20G nozzle. This test aimed to evaluate the hydrogel's ability to maintain structural integrity and continuity during and after the extrusion process.

##### 5.1.1.5 Self‐Healing and Adhesion

The self‐healing capabilities of the hydrogels were assessed through rheometry. To simulate damage, oscillatory shear strains of 100%, 300%, and 500% were applied for 60 s, exceeding the linear viscoelastic limit to ensure a breakdown of the network structure. Following damage, the strain was reduced to a small amplitude of 1%—within the linear viscoelastic region—to allow the network to self‐heal over a 60‐second period. The recovery of the hydrogel's mechanical properties was continuously monitored by measuring the storage modulus (G′) and loss modulus (G″) at a constant frequency of 1 Hz.^[^ ^78^ ^]^

During phonation, the vocal folds’ vibratory motion exerts forces from different directions on the microflap wound, including tension and shear loading. To evaluate the tensile adhesive strength, porcine larynges were bisected into two hemilarynges along the midsagittal line through the cricoid cartilage. The lamina propria and epithelial layers of the true vocal folds were delicately separated from the underlying vocalis muscle. Custom aluminum supporting blocks (8 × 10 × 50 mm) precoated with a thin layer of masking tape were used to mount the dissected tissues using super glue. The epithelial side of each fold was positioned in contact with the block, and 30 μL of hydrogel was uniformly applied to the lamina propria side before placing another tissue piece on top to form a bond (Figure S12b, Supporting Information). The bonded assemblies were allowed to cure for 60 min at 37 °C. After curing, the samples were tested for tensile strength using a universal traction test machine at a pull rate of 5 mm min^−1^ until failure occurred. Load, displacement, and time data were recorded to analyze the mechanical integrity of the adhesive bond.

The adhesion of hydrogels was also assessed using lap shear tests on porcine skin as the model tissue. A 500 μL aliquot of the hydrogel precursor was applied between two blocks with an overlapping bonding area of 20 mm × 10 mm. After 1 h curing time at 37 °C, the bonded samples were subjected to a tensile shear test using an Instron testing machine, operating at a crosshead speed of 5 mm min^−1^.

##### 5.1.1.6 Cell Culture

Immortalized human vocal fold fibroblasts (hVFFs) were cultured in Dulbecco's modified Eagle's medium (DMEM, Corning) supplemented with 10% fetal bovine serum (FBS) and 1% penicillin/streptomycin. Human monocytic THP‐1 cells were cultured in Roswell Park Memorial Institute (RPMI 1640) medium, supplemented with 10% FBS, 1% penicillin–streptomycin, 10 mM HEPES, and 1 mM sodium pyruvate. Cells were incubated at 37 °C in a humidified incubator containing 5% CO_2_, with the medium replaced every 3 days.

##### 5.1.1.7 Cytocompatibility and Cell Morphology

To assess the viability and morphology of hVFFs on hydrogels, 200 μL of cell‐free hydrogel precursors were pipetted into each well of an 8‐well μ‐slide (Ibidi, Martinsried, Germany) and allowed to cure for 1 h to form a thin layer. Subsequently, 200 μL of hVFF cell solution, at a concentration of 500 000 cells mL^−1^, was added to each well.

Cell viability within the hydrogels was evaluated using a live/dead viability/cytotoxicity kit (Invitrogen, US). After 72 h of incubation, cells were stained with calcein AM to mark live cells with green fluorescence and ethidium homodimer‐1 for dead cell labeling, emitting red fluorescence. Viability images were captured using a confocal laser scanning microscope (LSM 710, Zeiss, DE) at 10× magnification. Viability percentages were calculated by dividing the number of live cells by the total number of cells across multiple randomly selected fields to ensure statistical accuracy.

Detailed morphological data were obtained following a uniform 72‐h incubation period. Cells were fixed with 4% paraformaldehyde for 15 min, permeabilized with 0.1% Triton X‐100 for 10 min, and blocked with 1% BSA for 30 min. Cell cytoskeletons were stained with Alexa 633‐phalloidin for 45 min to visualize F‐actin filaments. Nuclei were stained with Hoechst 33 258 for 5 min. Fluorescent images were captured using an LSM 710 inverted confocal microscope at 10× magnification.

##### 5.1.1.8 Migration

Cell migration was analyzed using a modified Boyden Chamber experiment. A chemoattractant media gradient provided signaling cues to stimulate cell migration.^[^ ^79^ ^]^ This gradient was created by placing serum‐free DMEM in the upper chamber and DMEM supplemented with 10% FBS in the lower chamber. A volume of 200 μL of hydrogel precursor was transferred into Delta‐treated polycarbonate cell culture inserts with a pore size of 8 μm, which were then positioned within 12‐well plates. After the hydrogel was cured, 200 μL of vocal fold fibroblast suspension, at a seeding density of about 5 million cells/mL in serum‐free DMEM, was added to the hydrogel surface in each insert. These inserts were placed above 12‐well plates containing 400 μL of DMEM enriched with 10% FBS. To maintain the chemoattractant gradient, the DMEM + 10% FBS in the well plates was refreshed every 24 h over a week‐long incubation period. On the seventh day, the inserts were rinsed with PBS to remove nonmigrating cells. Migrated cells, adhering to the underside of the inserts and the wells, were detached using 200 μL of Trypsin‐EDTA (0.25% w/w). Gentle rocking was applied to ensure thorough dislodgment. After 2 min, enzymatic activity was halted by adding 800 μL of fresh DMEM + 10% FBS, and the resulting cell mixture was processed for flow cytometry analysis.

Migrated cells were enumerated using a FACSCantoII flow cytometer (BD Biosciences, CA). Prior to flow cytometric assessment, 50 μL of counting beads (Precision Count Beads, Biolegend, CA) at a concentration of 1.03 × 10^6^ particles/mL was added to each 500 μL cell aliquot. Given the distinctive size and granularity differences between the beads and fibroblasts, no additional staining was required. Each sample was processed in the flow cytometer until 2 × 10^4^ events were captured.

##### 5.1.1.9 Anti‐Inflammatory and Antifibrotic Effects of Curcumin: IF Staining and ELISA

To explore the potential antifibrotic effects of curcumin, hVFFs were seeded in a 24‐well plate at a density of 1 × 10^5^ cells per well. MDNC hydrogels were then placed in transwell inserts above the cells and incubated for 72 h. During the initial 24 h, cells were stimulated with 10 ng/mL TGF‐β1 to induce differentiation into myofibroblasts, evaluating curcumin's effectiveness in inhibiting this transformation.

The transformation of fibroblasts into myofibroblasts was evaluated by immunostaining for α‐smooth muscle actin (α‐SMA), a hallmark of the myofibroblastic phenotype, alongside collagen deposition. Postincubation, cells were fixed, permeabilized, and blocked to prepare for staining. They were then incubated overnight at 4 °C with primary antibodies against α‐SMA or type I collagen. Following this, cells were exposed to an Alexa 488‐conjugated secondary antibody for one hour at room temperature to enable fluorescence detection. Nuclei were counterstained with Hoechst 33 258 for 5 min to facilitate cell identification.^[^ ^80^ ^]^ Imaging was conducted using a confocal laser scanning microscope (LSM 710, Zeiss).

To investigate the potential of curcumin in inducing the anti‐inflammatory M2 phenotype, THP‐1 cells were seeded in a 24‐well plate at a density of 2 × 10^5^ cells per well. The cells were treated with 100 nM phorbol 12‐myristate 13‐acetate (PMA) for 48 h to induce differentiation into M0 macrophages. Hydrogels were then placed in transwell inserts above the differentiated cells and incubated for 72 h to assess curcumin's influence on macrophage polarization toward the M2 phenotype, which promotes wound healing and fibrosis prevention.

Postincubation, cells underwent fixation, permeabilization, and blocking to optimize antibody binding. They were then incubated overnight at 4 °C with primary CD206 antibodies, which specifically mark M2 macrophages. Subsequently, cells were treated with an Alexa 488‐conjugated secondary antibody for one hour at room temperature to facilitate fluorescence detection. Nuclei were counterstained with Hoechst 33 258 for 5 min, aiding in cell identification during confocal microscopy. Also, culture medium (supernatant) was collected and centrifuged to remove debris. An ELISA kit (Abcam) specific for interleukin‐10 (IL‐10) was used to quantify the levels of this anti‐inflammatory cytokine according to the manufacturer's instructions.

##### 5.1.1.10 Anti‐Inflammatory and Antifibrotic Effects of Curcumin: Gene Expression Analysis

Total RNA was extracted from cells using the Illustra RNA Spin Mini Kit (GE Healthcare, GE25‐0500‐71), following the manufacturer's instructions. The purity and concentration of RNA were assessed using a Nanodrop spectrophotometer (Thermo Fisher Scientific), accepting only samples with A260/A280 and A260/A230 ratios above 1.8 for further analysis. Subsequently, 500 ng of RNA was reverse transcribed using 5X RT Master Mix (Diamed, ABMG486) with the following incubation conditions: 10 min at 25 °C, 1 h at 42 °C, and 5 min at 85 °C to inactivate the enzyme. The synthesized cDNA was quantified using the Nanodrop to confirm efficient reverse transcription.

Quantitative PCR was performed to evaluate the expression levels of fibroblast and macrophage differentiation markers. The reactions utilized TaqMan Fast Master Mix (Thermo Fisher Scientific) and specific TaqMan probes for alpha‐SMA and COL1A1 for fibroblasts, and CD206 for THP‐1 cells. PCR amplification was carried out on a QuantStudio 7 Flex Real‐Time PCR system using the following thermal cycling conditions: an initial denaturation at 95 °C for 20 s, followed by 40 cycles of denaturation at 95 °C for 1 s, and annealing/extension at 60 °C for 20 s. Relative gene expression levels were determined using the ΔΔCt method, with glyceraldehyde‐3‐phosphate dehydrogenase serving as the endogenous control.

##### 5.1.1.11 In Vivo Assessment

All animal surgical procedures were reviewed and approved by the Facility Animal Care Committees at McGill University and the Research Institute of McGill University Health Centre (AUP‐MCGL‐8275). To evaluate the biocompatibility and regenerative potential of MDNC hydrogels compared to Restylane, a commercially available injectable hydrogel used for vocal fold applications, injections were performed on Sprague Dawley rats aged 5–20 weeks. Six rats were randomly assigned to receive either MDNC or commercial control (*n* = 3 per group), administered via subcutaneous injection in the dorsal region. Each rat received six injections under isoflurane anesthesia. Biocompatibility was assessed using the IVIS 100 (PerkinElmer). To evaluate the foreign body reactions, ProSense 680, a fluorescent probe sensitive to cathepsin activity, was injected 24 h before imaging sessions. Imaging was conducted on days 3, 7, and 21 to monitor the inflammatory response.

At the conclusion of the study period, the animals were euthanized, and the injected biomaterial and surrounding tissue were excised. The tissues were then fixed in 10% formalin, embedded in paraffin, cut into 5 mm sections, and stained using hematoxylin and eosin (H&E). This approach provided comprehensive insights into the inflammatory and healing responses elicited by the hydrogels, contributing to a thorough evaluation of their suitability for clinical applications in tissue regeneration.

##### 5.1.1.12 Statistical Analysis

All statistical analyses were performed using GraphPad Prism version 10.2.0 (GraphPad Software, Inc.). Statistical significance was determined using one‐way ANOVA followed by Tukey's Multiple Comparison Test. A *p*‐value of less than 0.05 was considered statistically significant. For analyses involving particle size and pore size, data from over 50 particles or pores were examined. All other characterizations were assessed in triplicate to ensure reproducibility. Error bars in graphical data represent the standard deviation of the mean. The significance levels are denoted as follows: not significant (ns); *p < 0.05; **p < 0.01; ***p < 0.001; ****p < 0.0001.

## Supporting Information

Supporting Information is available from the Wiley Online Library or from the author.

## Conflict of Interest

The authors declare no conflict of interest.

## Supporting information

Supplementary Material

## Data Availability

The data that support the findings of this study are available from the corresponding author upon reasonable request.
