# Supplementary material for: Multifunctional Double‐Network Hydrogel with Porous, Adhesive, and Immunomodulatory Properties for Minimally Invasive Soft Tissue Repair
Source: Small Sci. 2025 Oct 11;5(12):e202500368. doi: 10.1002/smsc.202500368 (PMC12697792; doi:10.1002/smsc.202500368)
Supplement: Supplementary file 1 — Supplementary Material [file SMSC-5-e202500368-s001.zip › smsc70125-sup-0001-SuppData-S1.pdf]

Supporting Information

Multifunctional Double-Network Hydrogel with Porous, Adhesive, and Immunomodulatory Properties for Minimally Invasive Soft Tissue Repair

Sara Nejati, Vahid Karamzadeh, Swen Groen, Malvika Nagrath, Luc Mongeau *

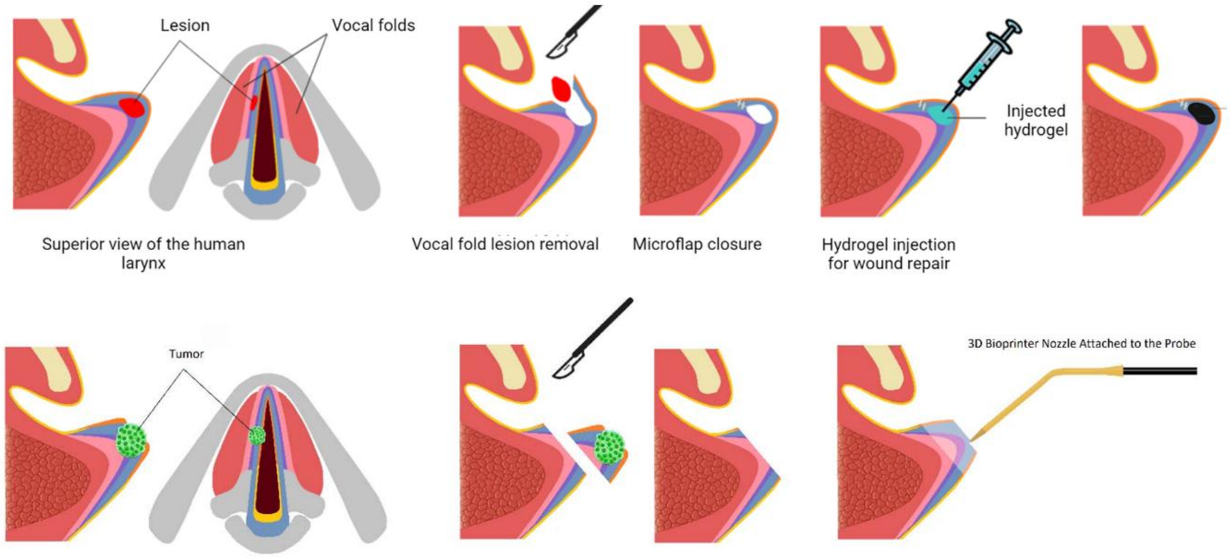

Figure S1. Schematic illustration showing the potential use of MDNC hydrogel for *in situ* vocal fold repair and regeneration.

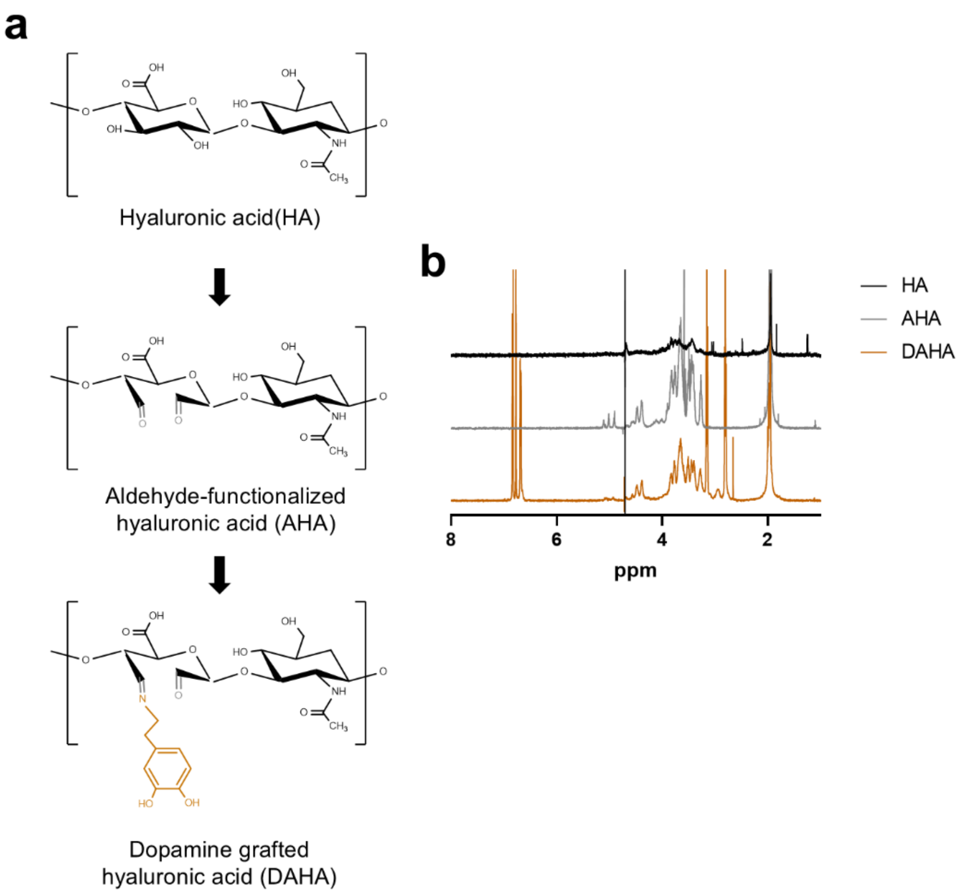

Figure S2. a) Schematic representation of the synthesis route for DAHA. b) ^{1}H NMR spectra of HA, AHA, and DAHA.

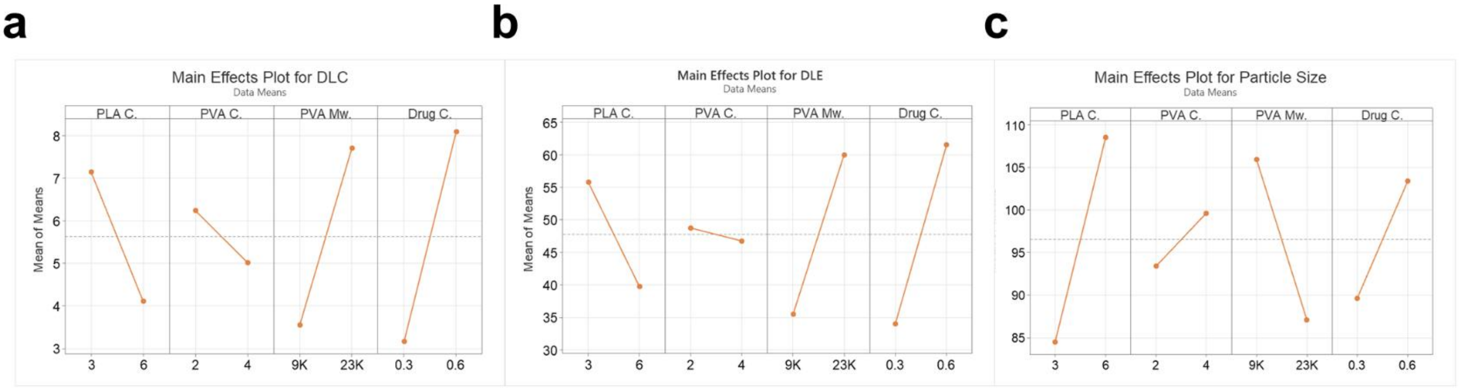

Figure S3. Main effects plots generated in Minitab, illustrating the impact of various formulation parameters on the characteristics of particles, specifically focusing on (a) particle size, (b) drug loading efficiency (DLE), and (c) drug loading content (DLC).

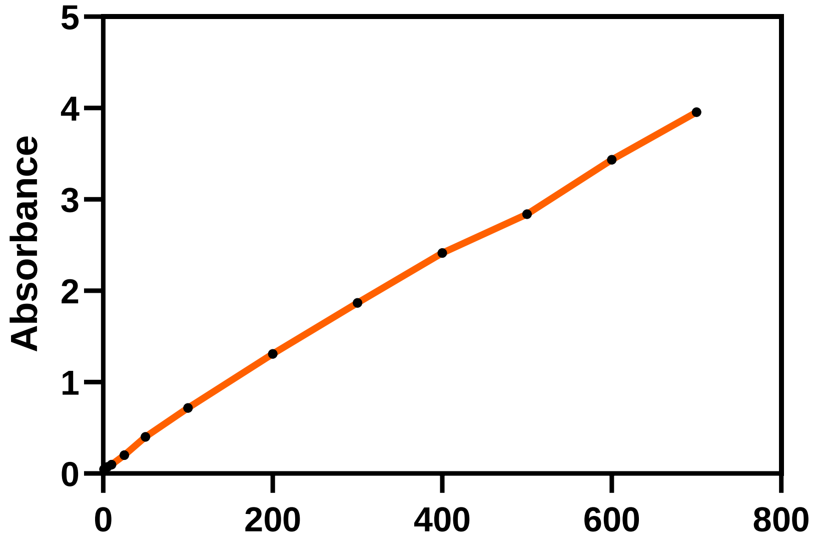

Figure S4. Calibration curve for curcumin, obtained by plotting the absorbance of the drug in the function of its concentration.

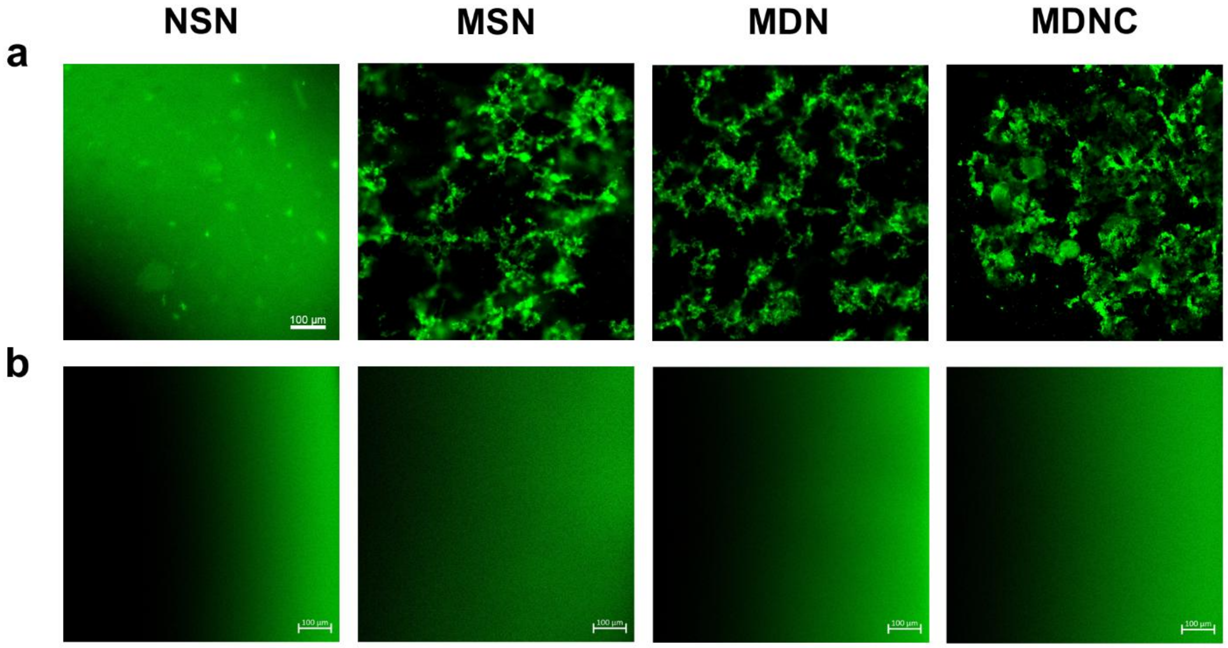

Figure S5. (a) Confocal microscopy images of FITC-labeled hydrogels showing the internal morphology in a hydrated state. (b) Confocal microscopy images capturing the progression of FITC dye diffusion into the hydrogels after 5 min. The dashed line indicates the border of the hydrogel with the dye solution.

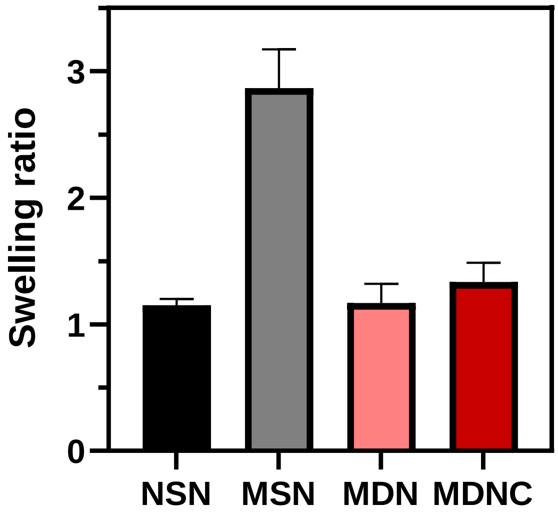

Figure S6. Swelling ratios of different hydrogels after 72 hours of immersion in PBS.

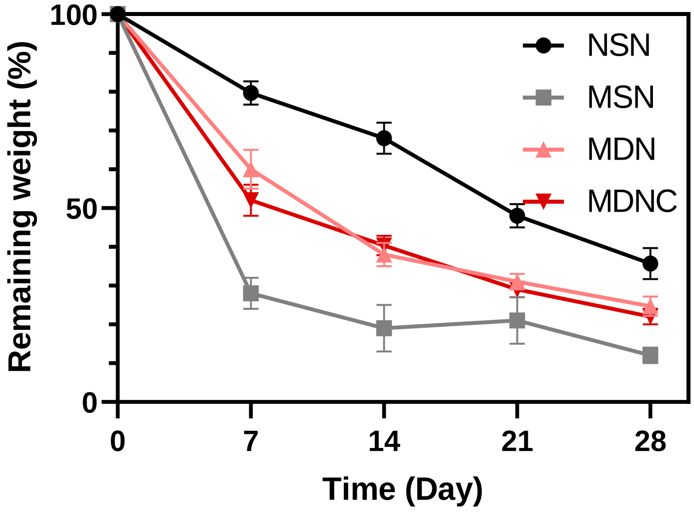

Figure S7. Degradation profile of hydrogels immersed in a lysozyme and hyaluronidase solution over a period of 4 weeks.

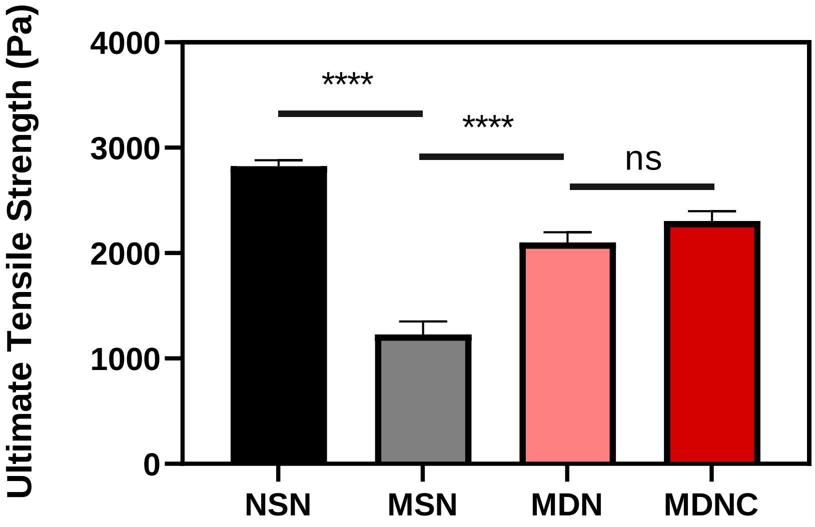

Figure S8. Ultimate tensile strength for each hydrogel formulation, derived from their respective stress-strain curves during tensile testing.

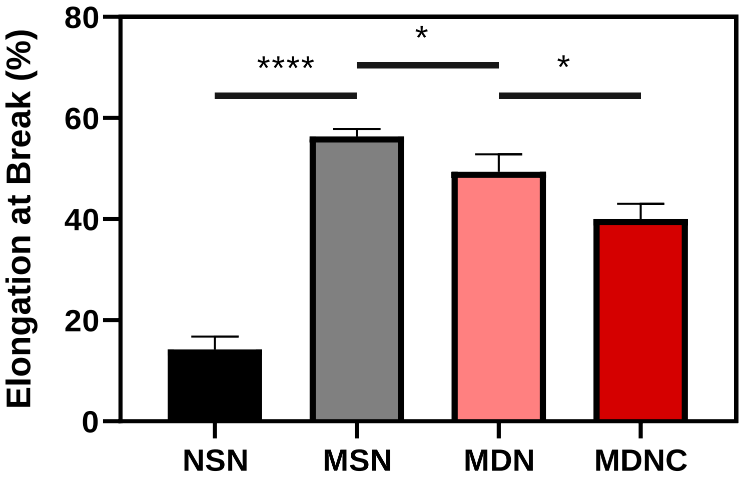

Figure S9. Elongation at break for different hydrogel formulations, derived from their stress-strain curves at the point of failure.

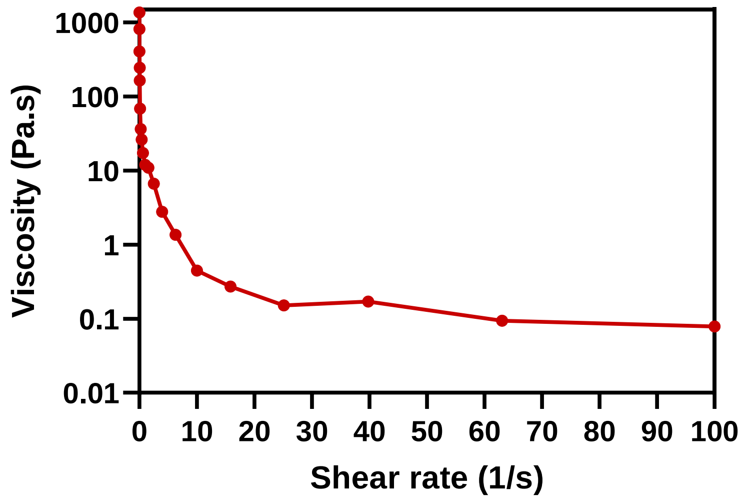

Figure S10. Viscosity curves depicting the shear-thinning behavior of the MDNC hydrogel, essential for injectability.

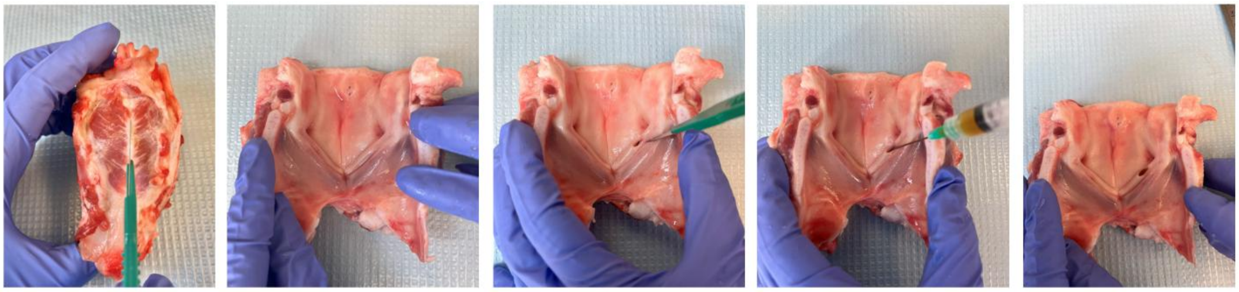

Figure S11. Injectability test of MDNC hydrogel into a void created in a procine vocal fold.

a

b

c

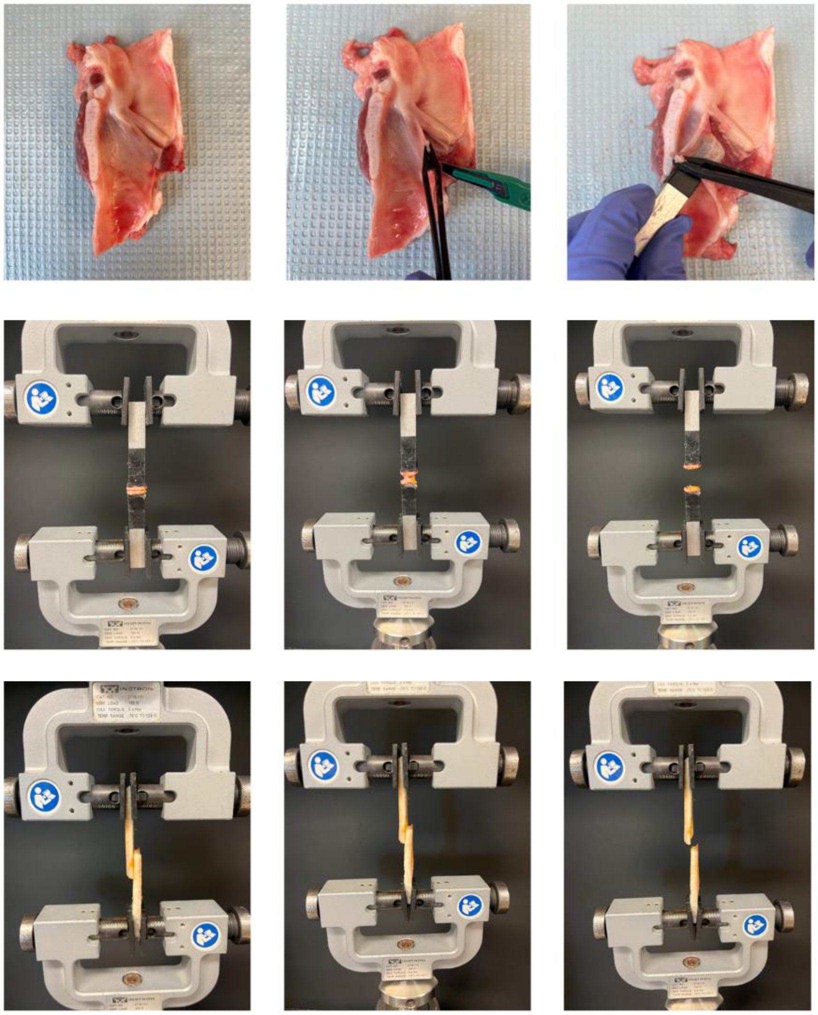

Figure S12. Adhesion Test Procedures for Hydrogels. (a) Dissection of porcine vocal fold tissue used for the tensile adhesion test. (b) Procedure for measuring tensile adhesion strength using an Instron machine. (c) Lap shear test procedure to assess shear adhesion strength on porcine skin.

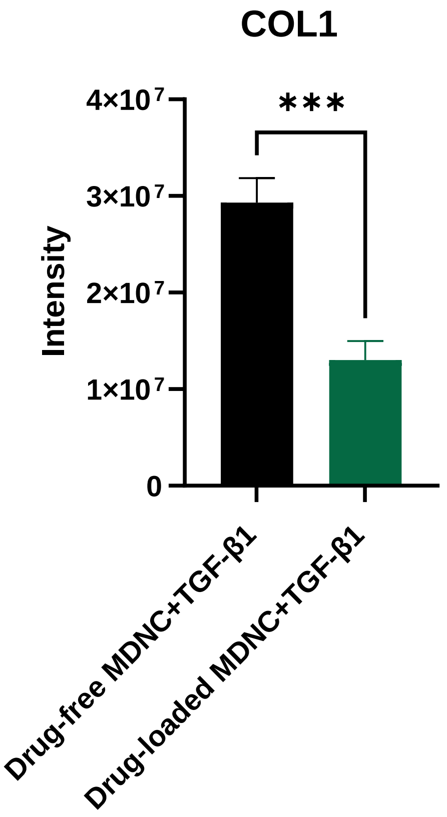

Figure S13. Quantification of Collagen-I production by the TGF-β1-treated hVFFs with and without curcumin treatment, derived from immunofluorescence-stained images.

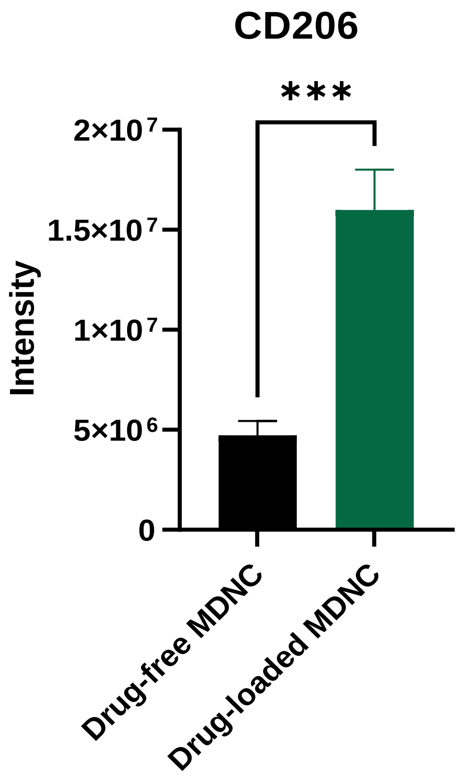

Figure S14. Quantification of CD206 expression by THP-1-derived macrophages with and without curcumin treatment, derived from immunofluorescence-stained images.

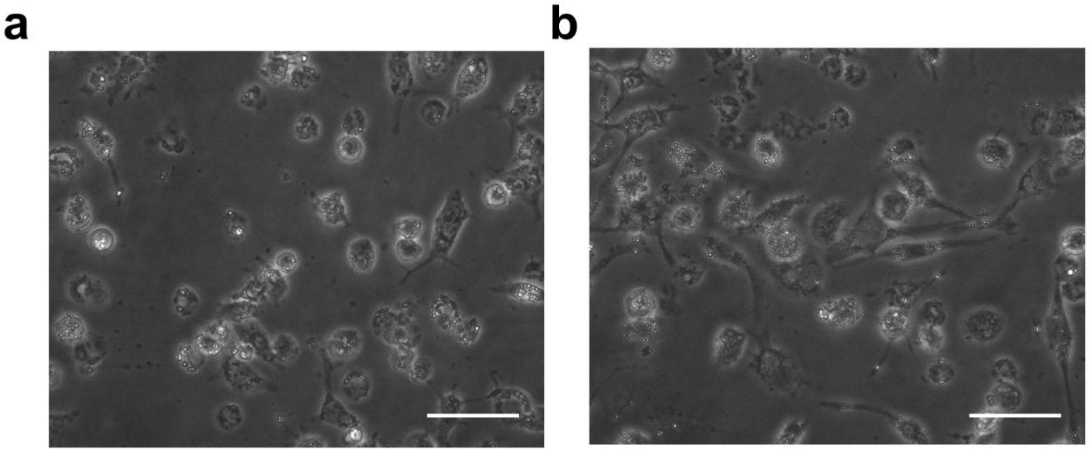

Figure S15. Light microscopy images showcasing the morphological differences in THP-1-derived macrophages with (right) and without (left) curcumin treatment.

Table S1. Comparative summary of hydrogel systems explored for vocal fold regeneration (2015–2025). NR indicates "not reported" in the source publication.

	Material System	Extrudability	Tissue Adhesion	Micro-porosity	Mechanical Matching	Intrinsic Bioactivity
**Mora-Navarro, 2026** [1]	VF lamina propria ECM hydrogel	Yes (injectable)	NR	NR	Stiffness matched to uninjured VF (1.84 mN)	Yes (antifibrotic, suppresses TGF-β/SMAD3)
**Nejati, 2024** [2]	Microporous GelMA/PEGDA	Yes (injectable)	NR	Yes (64.9–137.4 μm)	8.8–62.6 kPa tunable	NR
**John, 2024** [3]	Microgel composite (HA/ gelatin) + antifibrotic pirfenidone	Yes (injectable)	Yes (4–12 kPa)	Yes (30–80 μm)	~10–50 kPa (adjustable)	Yes (anti-fibrotic)
**Hamilton, 2023** [4]	Collagen I/III matrix	Yes (injectable)	Partial (matrix retention)	Fibrous, NR	NR	Yes (secreting ECM components)
**Zou, 2023** [5]	Self-fusing CMCS/alginate	Yes (injectable)	NR	Fibrous, not explicitly microporous	Matched VF function, durable	Yes (ECM-inducing bioactivity)
**Nejati, 2023** [6]	DAHA/HAMA double-network	Yes (injectable)	Yes (~12.8 kPa)	Yes (~27 μm pores)	E ~ 29.9 kPa	NR
**Ng et al., 2023** [7]	Genipin-crosslinked gelatin hydrogel	Yes (injectable)	NR	Yes (~100–400 μm)	E ~ 2–10 kPa	NR

Table S1. Cont.

	Material System	Extrudability	Tissue Adhesion	Micro-porosity	Mechanical Matching	Intrinsic Bioactivity
**Song, 2022** [8]	HA-Tz/ Nb-TCO-tagged bioactive peptides	Yes (injectable)	NR	Yes, ~40–80 µm	2–10 kPa	Yes (regulates myofibroblast)
**Mohammadi, 2022** [9]	GelMA/ HAMA microgels + IL-10	Yes (injectable)	NR	Yes (~70–120 µm)	E ~ 2-6 kPa	Yes (IL-10 anti-fibrotic, pro-M2)
**Hu, 2022** [10]	HA-waterborne polyurethane hydrogel	Yes (injectable)	Yes (~6.5 kPa)	NR	G′ ~2.7–5.2 kPa	Yes (scarless healing, anti-fibrotic)
**Bao, 2021** [11]	Chitosan/ Glycol-chitosan PDN	Yes (injectable)	NR	Yes (~ 6-10 µm)	E ~ 3.5–9 kPa (resilient, >6 million cycles at 120 Hz)	Partial (antimicrobial)
**Pruett, 2019** [12,13]	MAP hydrogel (PEG microgels)	Yes (injectable)	NR	Yes (50–100 µm)	G′ ~5–6 kPa	NR
**Hertegård, 2019** [14]	HA hydrogel + hMSC	Yes (injectable)	NR	NR	E ~ 400 - 1,500 Pa	Yes (immunomodulation via MSCs)
**Ravanbakhsh, 2019** [15]	CNT–glycol chitosan hydrogel	Yes (injectable)	NR	Yes (pore size NR, ↑120% with CNTs)	E ~ 10–30 Pa; tunable	NR

Table S1. Cont.

	Material System	Extrudability	Tissue Adhesion	Micro-porosity	Mechanical Matching	Intrinsic Bioactivity
**Walimbe, 2019** [16]	Thiolated HA + PEGDA + Col I/III	Yes (injectable)	NR	Yes (pore size NR)	Tunable; increased by collagen	NR
**King, 2019** [17]	RLP/HA hybrid hydrogel	Yes (injectable)	NR	NR	G′ ~ 600 Pa	NR
**Kim, 2019** [18]	Plasmid-DNA-loaded hydrogel (PEI/HA/PEG)	Yes (injectable)	NR	NR	NR	Yes (gene delivery)
**Brown, 2018** [19]	Silk microparticles in HA	Yes (injectable, catheter/needle)	NR	Yes (10–100 μm)	E ~ 1–3 kPa	NR
**Li, 2018** [20]	Resilin-based hydrogel	Yes (injectable)	NR	NR	E ~ 1–2 kPa	NR
**Erndt-Marino, 2017** [21]	bFGF-HA hydrogel	Yes (injectable)	NR	NR	NR	Yes (bFGF delivery, pro-ECM)
**Imaizumi, 2017** [22]	Injectable HA hydrogel + hiPSC	Yes (injectable)	NR	NR	E ~ 400–1,500 Pa	Yes (cell delivery)

Table S1. Cont.

	Material System	Extrudability	Tissue Adhesion	Micro-porosity	Mechanical Matching	Intrinsic Bioactivity
**Kobayashi, 2017** [23]	Gelatin hydrogel (bFGF delivery)	Yes (injectable)	NR	NR	NR	Yes (pro-ECM, bFGF)
**Huang, 2016** [24]	HA + Acellular matrix cogels	Yes (injectable)	NR	NR	NR	Yes (ECM cues)
**Kazemirad, 2015** [25]	HA-Gelatin hydrogel	Yes (injectable)	NR	NR	G′ ~500 Pa	NR
**Lim et al., 2015** [26]	β-Glucan hydrogel cross-linked by γ-irradiation	Yes (injectable)	NR	NR	NR	NR

References

[1] C. Mora-Navarro, E. Smith, Z. Wang, M. del C. Ramos-Alamo, L. Collins, N. Awad, D. R. D. Cruz, T. S. Tollison, I. Huntress, G. Gartling, R. Nakamura, G. R. Dion, X. Peng, R. C. Branski, D. O. Freytes, *Biomaterials Advances* **2026**, *178*, 214424.

[2] S. Nejati, L. Mongeau, *ACS Biomater Sci Eng* **2024**, *10*, 3909.

[3] M. John, A. Nabizath, S. Krishnakumar, U. Menon, D. Menon, M. Nair, *ACS Appl Bio Mater* **2024**, *7*, 5237.

[4] N. J. I. Hamilton, A. Tait, B. Weil, J. Daniels, *Laryngoscope* **2024**, *134*, 882.

[5] C. Y. Zou, J. J. Hu, D. Lu, Q. J. Li, Y. L. Jiang, R. Wang, H. Y. Wang, X. X. Lei, J. Li-Ling, H. Yang, H. Q. Xie, *Bioact Mater* **2023**, *24*, 54.

[6] S. Nejati, L. Mongeau, *Sci Rep* **2023**, *13*, 1.

[7] W. C. Ng, Y. Lokanathan, M. B. Fauzi, M. M. Baki, A. A. Zainuddin, S. J. Phang, M. Azman, *Sci Rep* **2023**, *13*, 1.

[8] J. Song, H. Gao, H. Zhang, O. J. George, A. S. Hillman, J. M. Fox, X. Jia, *ACS Appl Mater Interfaces* **2022**, *14*, 51669.

[9] S. Mohammadi, H. Ravanbakhsh, S. Taheri, G. Bao, L. Mongeau, *Adv Healthc Mater* **2022**, *11*.

[10] J. J. Hu, M. Wang, X. X. Lei, Y. L. Jiang, L. Yuan, Z. J. Pan, D. Lu, F. Luo, J. H. Li, H. Tan, *ACS Appl Mater Interfaces* **2022**, *14*, 42827.

[11] S. Taheri, G. Bao, Z. He, S. Mohammadi, H. Ravanbakhsh, L. Lessard, J. Li, L. Mongeau, *Advanced Science* **2022**, *9*, 1.

[12] L. Pruett, H. Koehn, T. Martz, I. Churnin, S. Ferrante, L. Salopek, P. Cottler, D. R. Griffin, J. J. Daniero, *Laryngoscope* **2020**, *130*, 2432.

[13] L. J. Pruett, H. L. Kenny, W. M. Swift, K. J. Catallo, Z. R. Apsel, L. S. Salopek, P. O. Scumpia, P. S. Cottler, D. R. Griffin, J. J. Daniero, *NPJ Regen Med* **2023**, *8*, 1.

[14] S. Hertegård, S. R. Nagubothu, E. Malmström, C. E. Ström, A. Tolf, L. C. Davies, K. Le Blanc, *Stem Cells Dev* **2019**, *28*, 1177.

[15] H. Ravanbakhsh, G. Bao, N. Latifi, L. G. Mongeau, *Materials Science and Engineering C* **2019**, *103*.

[16] T. Walimbe, S. Calve, A. Panitch, M. P. Sivasankar, *Acta Biomater* **2019**, *87*, 97.

[17] R. E. King, H. K. Lau, H. Zhang, I. Sidhu, M. B. Christensen, E. W. Fowler, L. Li, X. Jia, K. L. Kiick, S. L. Thibeault, *Regen Eng Transl Med* **2019**, *5*, 373.

[18] I. G. Kim, M. R. Park, Y. H. Choi, J. S. Choi, H. J. Ahn, S. K. Kwon, J. H. Lee, *ACS Biomater Sci Eng* **2019**, *5*, 1497.

[19] J. E. Brown, C. P. Gulka, J. E. M. Giordano, M. P. Montero, A. Hoang, T. L. Carroll, *Journal of Voice* **2019**, *33*, 773.

[20] L. Li, J. M. Stiadle, E. E. Levendoski, H. K. Lau, S. L. Thibeault, K. L. Kiick, *J Biomed Mater Res A* **2018**, *106*, 2229.

[21] J. D. Erndt-Marino, A. C. Jimenez-Vergara, P. Diaz-Rodriguez, J. Kulwatno, J. F. Diaz-Quiroz, S. Thibeault, M. S. Hahn, *J Biomed Mater Res B Appl Biomater* **2018**, *106*, 1258.

[22] M. Imaizumi, N. Y. K. Li-Jessen, Y. Sato, D. T. Yang, S. L. Thibeault, *Annals of Otology, Rhinology and Laryngology* **2017**, *126*, 304.

[23] T. Kobayashi, M. Mizuta, N. Hiwatashi, Y. Kishimoto, T. Nakamura, S. ichi Kanemaru, S. Hirano, *Auris Nasus Larynx* **2017**, *44*, 86.

[24] D. Huang, R. Wang, S. Yang, *Biomed Res Int* **2016**, *2016*.

[25] S. Kazemirad, H. K. Heris, L. Mongeau, *J Biomed Mater Res B Appl Biomater* **2016**, *104*, 283.

[26] Y. M. Lim, B. H. Kim, H. B. Kim, E. Park, S. W. Park, J. S. Park, S. I. Choi, T. K. Kwon, S. K. Kwon, *Biomed Res Int* **2015**, *2015*.
